# A common arthropod from the Late Ordovician Big Hill Lagerstätte (Michigan) reveals an unexpected ecological diversity within Chasmataspidida

**DOI:** 10.1186/s12862-018-1329-4

**Published:** 2019-01-08

**Authors:** James C. Lamsdell, Gerald O. Gunderson, Ronald C. Meyer

**Affiliations:** 1 Department of Geology and Geography, West Virginia University, 98 Beechurst Avenue, Brooks Hall, Morgantown, WV 26501 USA; 2 Middleton, USA; 3 Louiseville, USA

**Keywords:** *Hoplitaspis*, Chasmataspidida, Big Hill, Diploaspididae, Ordovician, Lagerstätte, Chelicerata, Microtergite, Podomere 7a, Swimming paddle

## Abstract

**Background:**

Chasmataspidids are a rare group of chelicerate arthropods known from 12 species assigned to ten genera, with a geologic range extending from the Ordovician to the Devonian. The Late Ordovician (Richmondian) fauna of the Big Hill Lagerstätte includes a new species of chasmataspidid represented by 55 specimens. This taxon is only the second chasmataspidid described from the Ordovician and preserves morphological details unknown from any of the previously described species.

**Results:**

The new chasmataspidid species is described as *Hoplitaspis hiawathai* gen. et sp. nov.. Comparison with all other known chasmataspidids indicates that *Hoplitaspis* occupies an intermediate morphological position between the Ordovician *Chasmataspis* and the Silurian-Devonian diploaspidids. While the modification of appendage VI into a broad swimming paddle allies *Hoplitaspis* to the Diploaspididae, the paddle lacks the anterior ‘podomere 7a’ found in other diploaspidids and shows evidence of having been derived from a *Chasmataspis*-like chelate appendage. Other details, such as the large body size and degree of expression of the first tergite, show clear affinities with *Chasmataspis*, providing strong support for chasmataspidid monophyly.

**Conclusions:**

The large body size and well-developed appendage armature of *Hoplitaspis* reveals that chasmataspidids occupied a greater breadth of ecological roles than previously thought, with the abundance of available specimens indicating that *Hoplitaspis* was an important component of the local community. The miniaturization and ecological limiting of diploaspidids potentially coincides with the major radiation of eurypterids and may suggest some degree of competition between the two groups. The geographic distribution of chasmataspidid species suggests the group may have originated in Laurentia and migrated to the paleocontinents of Baltica and Siberia as tectonic processes drew the paleocontinents into close proximity.

## Background

Chasmataspidids are a rare group of Paleozoic chelicerate arthropods. Known from only 12 species assigned to 10 genera [1], chasmataspidids are characterized by the possession of a fused opisthosomal buckler and their typically diminutive (< 30 mm) size. Chelicerate phylogeny indicates that the group is sister to a clade comprising eurypterids and arachnids [2–4], although poor preservation of a number of species has hindered attempts at resolving chasmataspidid internal relationships. Currently, chasmataspidids are divided between two family groups: Chasmataspididae, comprising only the species *Chasmataspis laurencii* from the Early Ordovician of Tennessee [5, 6]; and Diploaspididae, encompassing all other known species from the Silurian to Devonian of Europe, North America, and Russia [1, 7]. Chasmataspidids reach their acme in the Early Devonian, a period from which the majority of species are known. Only two species of diploaspidid are known from the Silurian, *Loganamaraspis dunlopi* and *Diploaspis praecursor* [1, 7] and these, along with *Chasmataspis*, form the entirety of the pre-Devonian chasmataspidid fossil record. *Chasmataspis* is morphologically distinct from the other chasmataspidids, being considerably larger than the majority of diploaspidids with a broad semicircular carapace and chelate prosomal appendages. As it currently stands, a 40 million year gap in the fossil record exists between *Chasmataspis* in the Early Ordovician and the next known chasmataspidid, *Loganamaraspis*, in the Early Silurian.

Here, we describe a new species of diploaspidid chasmataspidid, *Hoplitaspis hiawathai* gen. et. sp. nov., from the Late Ordovician (late Katian, Richmondian; 449–445 Ma) Big Hill Lagerstätte of Stonington Peninsula, Michigan [8], extending the stratigraphic range of Diploaspididae back some 12 million years. The material is abundant and exceptionally preserved, providing remarkably complete information about the overall morphology. The majority of specimens preserve some degree of three-dimensional preservation including some internal cuticular structures, permitting dissection through the fossilized organisms.

This new taxon is important in expanding our limited knowledge on detailed chasmataspidid morphology. The majority of chasmataspidid species are known from single, poorly preserved specimens [1, 7, 9, 10], with specimens displaying articulated appendages known only from the Devonian [11, 12]. *Hoplitaspis* also fills the temporal gap between *Chasmataspis* and the remaining diploaspidids. Here we describe the new species and discuss its significance for the early evolution of chasmataspidids.

## Methods

### Material

The specimens described here were collected from the upper 24 cm of the 3.2 m section of the Big Hill Formation exposed at Stonington Peninsular in Michigan’s Upper Peninsula [8] collected between 2013 and 2016. Permission to excavate at the Stonington Peninsular locality was given by the Board of Commissioners of Delta County, Michigan. Most specimens were excavated directly from the outcrop and have a dull cream color, with a few additional specimens collected from eroded-out blocks that have weathered to a light grey. The locality has so far yielded over 400 individual organisms on 232 accessioned specimens, of which about 13.7% are chasmataspidid remains. Arthropods, which also include leperditids (9.5%), eurypterids (3.7%), and rare trilobites (0.5%) and xiphosurans (0.2%) make up the most diverse invertebrate group of the Big Hill biota, although medusae (25.6%) and brachiopods (21.7%) dominate (Table 1). All material described here is accessioned in the University of Wisconsin Geology Museum (UWGM).

**Table 1:** Composition of the Big Hill biota

Component of biota	Number of specimens	Percentage of biota
Alga	67	16.6
Microbial mat	2	0.5
Medusa	103	25.6
Poriferan	4	1.0
Halloporid bryozoan	2	0.5
Discinoid brachiopod	13	3.3
Linguloid brachiopod	74	18.4
Bivalve	5	1.2
Gastropod	5	1.2
Cephalopod	11	2.7
Trilobite	2	0.5
Leperditid	38	9.5
Chasmataspidid	55	13.7
Eurypterid	15	3.7
Xiphosuran	1	0.2
Conodont element	1	0.2
Carbonaceous tube	5	1.2

Specimens were photographed using a Canon EOS 60D digital camera with a Canon EF-S 60 mm f/2.8 Macro USM lens. All specimens were imaged dry and with normal light. Additionally, fine details of some specimens were imaged immersed in ethanol. Image cropping and leveling was carried out using Adobe Photoshop CC 2015, and figures were prepared with Adobe Illustrator CC 2015, on a Microsoft Surface Studio running Windows 10.

### Geological setting and preservation

The Big Hill Formation is a 39 m thick [13] component of the Richmond Group, of which the lowermost 3–4 m are exposed at the Stonington Peninsula locality [14]. The formation is late Katian (Richmondian; 449–445 Ma) in age, overlying the Stonington Formation and in turn discomformably overlain by the Late Ordovician–Silurian (Hirnantian–Llandovery) Manitoulin Dolomite [15].

**Fig. 1:**
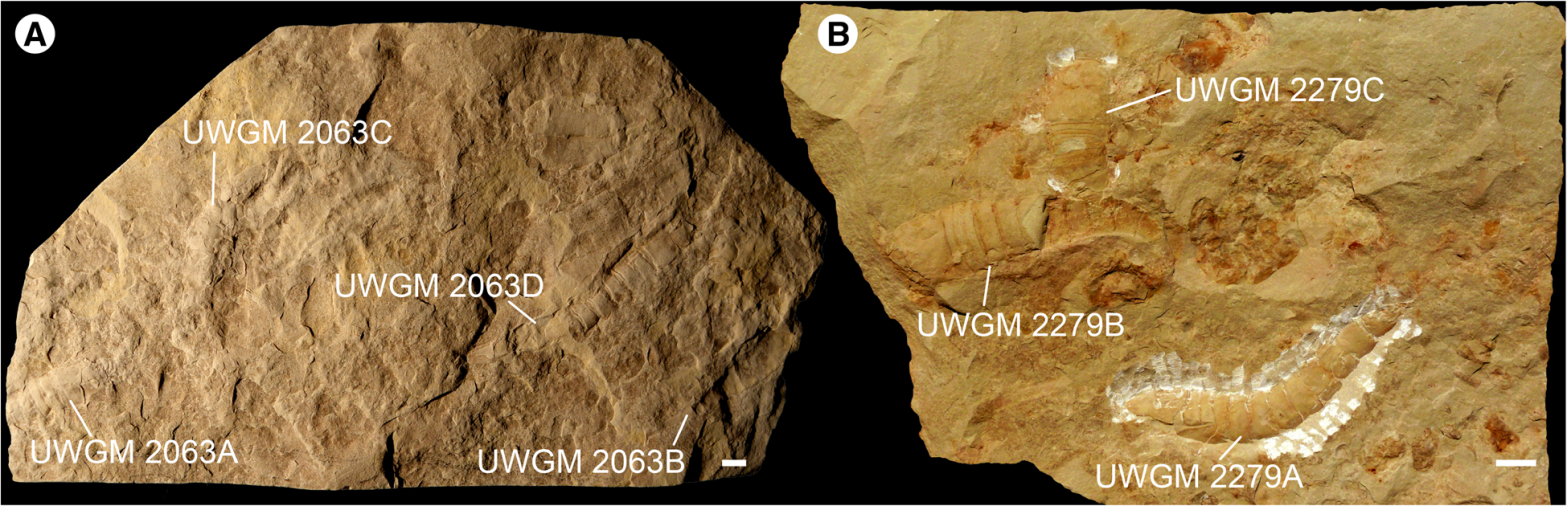
*Hoplitaspis hiawathai*, blocks preserving multiple specimens. **a** UWGM 2063, preserving at least four individuals. **b** UWGM 2279, preserving three individuals. Scale bars = 10 mm

The lithology of the Big Hill Formation comprises grey dolomitic mudstones at its very base that are rapidly replaced by fine-grained dolostones, with occasional bands of shale, that make up the majority of the formation [16]. The exceptionally-preserved biota is located in a 24 cm thick interval of the Big Hill Formation characterized by thinly bedded fine-grained dolostone beds and green-brown dolomitic shales [8]. The fossils occur in accumulations within shallow troughs on the upper surfaces of the dolostone beds, where they are overlain by shale. These troughs likely represent the original sea-floor topology, with the organisms accumulating within the depressions over time. As such, multiple specimens are frequently found in close association with one another (Fig. 1). Occasional exceptionally preserved fossils also occur within the dolostone and, rarely, in the dolomitic shale.

The environment of deposition is considered to have been a shallow, restricted lagoon in marginal marine settings [8]. Abundant intact algal specimens suggesting in situ burial indicate the lagoon floor was within the photic zone, although the lack of current or wave ripples on bedding surfaces suggests a depth of several meters. The depauperate biota may indicate a dysoxic setting under non-standard marine salinity, a possibility supported by the abundance of lingulid brachiopods and absence of rhynchonelliform brachiopods and echinoderms, although sedimentological evidence is lacking. Some interchange with fully marine settings clearly occurred, however, as indicated by the accumulation of large numbers of medusae exhibiting different stages of decay and orthocones possibly representing empty conchs that drifted into the environment. The fact that these carcasses were able to accumulate undisturbed on the sediment surface for long periods before burial attests to the low energy setting of the environment as well as the absence of large numbers of predators or scavengers. The chelicerates, including the chasmataspidids, also likely entered the lagoon from the open marine realm as indicated by the absence of carcasses (see below), which would be expected to be present if the animals were spending extended periods of time within the lagoon.

While clearly predatory, the chasmataspidids likely did not enter the lagoon to feed upon the biological detritus, rather they appear to have taken advantage of the sheltered environment to molt. A number of characteristics indicate that the chasmataspidid specimens are exuviae, including the curvature of the opisthosoma and outstretched position of the prosomal limbs, which are characteristics shared with modern scorpion molts [17]. The preabdominal buckler is frequently dorso-ventrally disarticulated, a characteristic associated with exuviae of the Devonian *Diploaspis casteri* [18], and there is no indication of organic staining around the specimens as would be expected from internal soft-tissue decay [19]. The high degree of articulation exhibited by the specimens indicates that they were buried soon after ecdysis in a low energy environment [20]. The occurrence of the biota on the dolomite bedding surfaces suggests that preservation was likely facilitated by the influx of silt due to either continental runoff after extreme rainfall or swamping of the lagoon by marine sediment thrown up during storm events. The completeness of the alga and chasmataspidids would seem to indicate that a continental source is more likely, as wave energy generated by extreme weather conditions would have broken up the specimens. However, it is currently impossible to state with any certainty that the silt deposition was the result of a continental rather than marine event. The repeated occurrence of the biota on the bedding surfaces indicates that the lagoonal environment was otherwise generally stable, and that without the sudden burial normal decay processes operated resulting in the complete breakdown of biological material.

Smaller chasmataspidid specimens are preserved as flattened compressions on the bedding plane surface and were clearly somewhat pliable at the time of burial as shown by the manner in which some specimens are superimposed in part over three-dimensionally preserved leperditid valves. Larger individuals are occasionally preserved in three dimensions, with the interior of the specimens filled with the same dolomitic shale as overlies the dolostone bedding planes. It is thought that silt entering the lagoon infiltrated some of the larger exuviae and filled the internal void space, which would then have resisted compression from subsequently deposited overlying sediments. The surfaces of all specimens are darker than the surrounding matrix, although the original cuticle is absent except for when the coxal gnathobases are present, where it occurs on the gnathobasic spines as a dark brown film. Vestiges of cuticle are also observed on some of the smaller specimens where the cuticular ornament can be seen as a red-brown stain. Ornamentation is otherwise absent on all other specimens, and no lens structures are observed in any of the preserved lateral eyes. Limbs, where present, are generally intact. A number of specimens exhibit splitting of the dorsal and ventral components of the buckler, a condition that apparently occurred during molting in chasmataspidids [18]. Somewhat more common is the loss of the dorsal prosomal shield (the carapace), which also frequently occurs in eurypterid exuviae [20]. Another common point of disarticulation appears to have been the connection between the buckler and the postabdomen, as several specimens comprise only the freely-articulating 5th–13th opisthosomal segments and telson.

### Institutional abbreviations

MM, Manitoba Museum, Winnipeg, Manitoba, Canada.

UWGM, University of Wisconsin Geology Museum, Madison, WI, USA.

### Terminology

Chasmataspidid morphological terminology follows Tetlie and Braddy [7], who largely adopted Tollerton’s [21] terms for eurypterid terminology relating to the shape of the carapace, lateral eye shape and position, and metastoma shape. Terminology for prosomal structures and the labeling of appendages follows the standards set out for eurypterids by Selden [22]. Minor modifications to the terminology used in these papers follows Lamsdell [23]. Terminology for morphology apparently unique to chasmataspidids, such as the preabdominal buckler composed of opisthosomal segments 2–4 and comprising a dorsal and ventral shield, is derived from Caster and Brooks [5] and Størmer [24], with additions from Marshall et al. [11].

### Nomenclatural acts

This article conforms to the requirements of the amended International Code of Zoological Nomenclature, and hence the new names contained herein are available under that Code. This published work and the nomenclatural acts it contains have been registered in ZooBank, the online registration system for the ICZN. The ZooBank LSIDs (Life Science Identifiers) can be resolved and the associated information viewed through any standard web browser by appending the LSID to the prefix “http://zoobank.org”. The LSID for this publication is: urn:lsid:zoobank.org:pub:4A1B411A-9C4E-4141-B4CD-AA0B58A8ECF9. The journal is identified by ISSN 1471–2148, and has been archived and is available from the following digital repositories: PubMed Central, LOCKSS, INIST, and Koninklijke Bibliotheek.

## Results

### Systematic paleontology

CHELICERATA Heymons, 1901

EUCHELICERATA Weygoldt and Paulus, 1979

CHASMATASPIDIDA Caster and Brooks, 1956

DIPLOASPIDIDAE Størmer, 1972

#### Remarks

The family Diploaspididae is currently diagnosed based on the possession of a preabdomen with curved, non-trilobate segments, a tapering postabdomen, and a short telson [11]. *Hoplitaspis* does not perfectly fit this diagnosis, as it possesses relatively straight preabdominal segments and a long telson. In general morphology it shares many commonalities with *Loganamaraspis* [7] and *Dvulikiaspis* [11], both of which are currently considered diploaspidids. As it is unclear whether these three species form a clade it is currently considered inappropriate to erect a new family, and so *Hoplitaspis* is also accommodated within Diploaspididae pending a wider revision of the group.

*Hoplitaspis* gen. nov

LSID: *urn:lsid:zoobank.org:act:68E6F452-964D-4902-A975-4AEF71FA7652*

#### Etymology

The genus is named after the hoplite (Greek όπλίτης), the citizen-soldier of Ancient Greek city-states armed with spears and shields that fought in the phalanx formation, presenting an array of spears towards their foes in a manner that is superficially similar to the appendage armature of *Hoplitaspis*. This is combined with -aspis (άσπίς – shield), the epithet typically applied to chasmataspidid genera.

#### Diagnosis

Diploaspidid with subquadrate to horseshoe-shaped prosomal shield; ovoreniform lateral eyes positioned intermediate between antelateral and centrilateral; prosomal appendages II–V bearing paired elongated spines on all but the penultimate podomere (equivalent to the eurypterid *Carcinosoma-*type appendage); prosomal appendage VI composed of eight podomeres, distally expanded into a paddle; metastoma obovate; first tergite only slightly reduced in length; weakly expressed buckler with little first-order differentiation, tergites lacking extreme curvature; postabdomen long, accounting for *c.* 75% of the total body length; pretelson elongated; telson narrow, lanceolate.

*Hoplitaspis hiawathai* sp. nov.

LSID: *urn:lsid:zoobank.org:act:DF752F67-AE9D-4BD1-9EE8-70A0-E8D02CC7*

Figures 1, 2, 3, 4, 5, 6, 7, 8, 9, 10, 11 and 12.

**Fig. 2:**
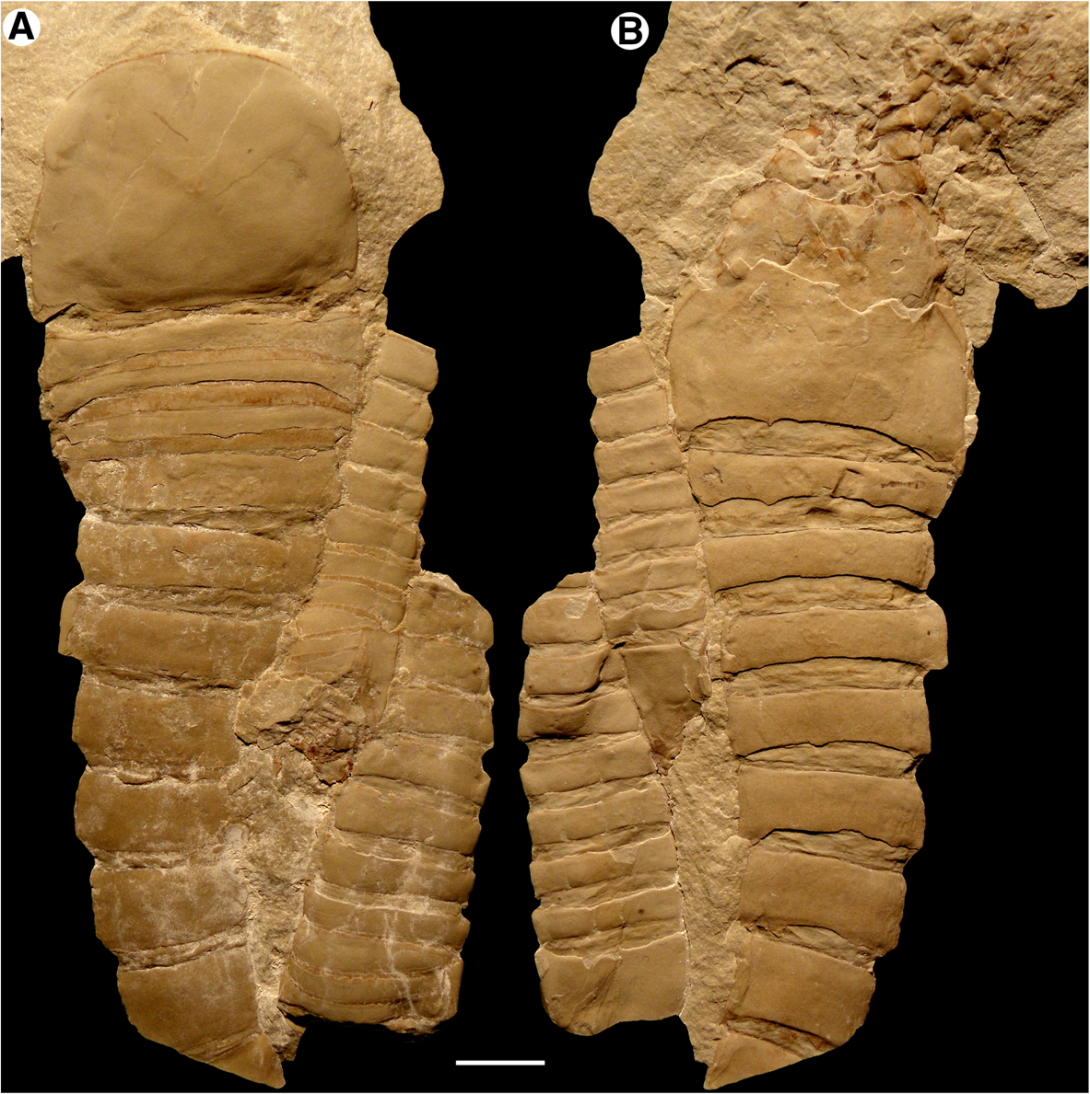
*Hoplitaspis hiawathai*, UWGM 1873 – three almost complete specimens. **a** Dorsal view of UWGM 1873A (holotype; largest specimen), preserving prosomal shield, buckler, and postabdomen. Note the ventral buckler plate underlying tergite 2–4 in UWGM 1873B (central specimen). UWGM 1873C, displaying the buckler and seven postabdominal segments, is on the right. **b** Ventral view of UWGM 1873A, showing the coxa, metastoma, and ventral buckler plate. Scale bar = 10 mm

**Fig. 3:**
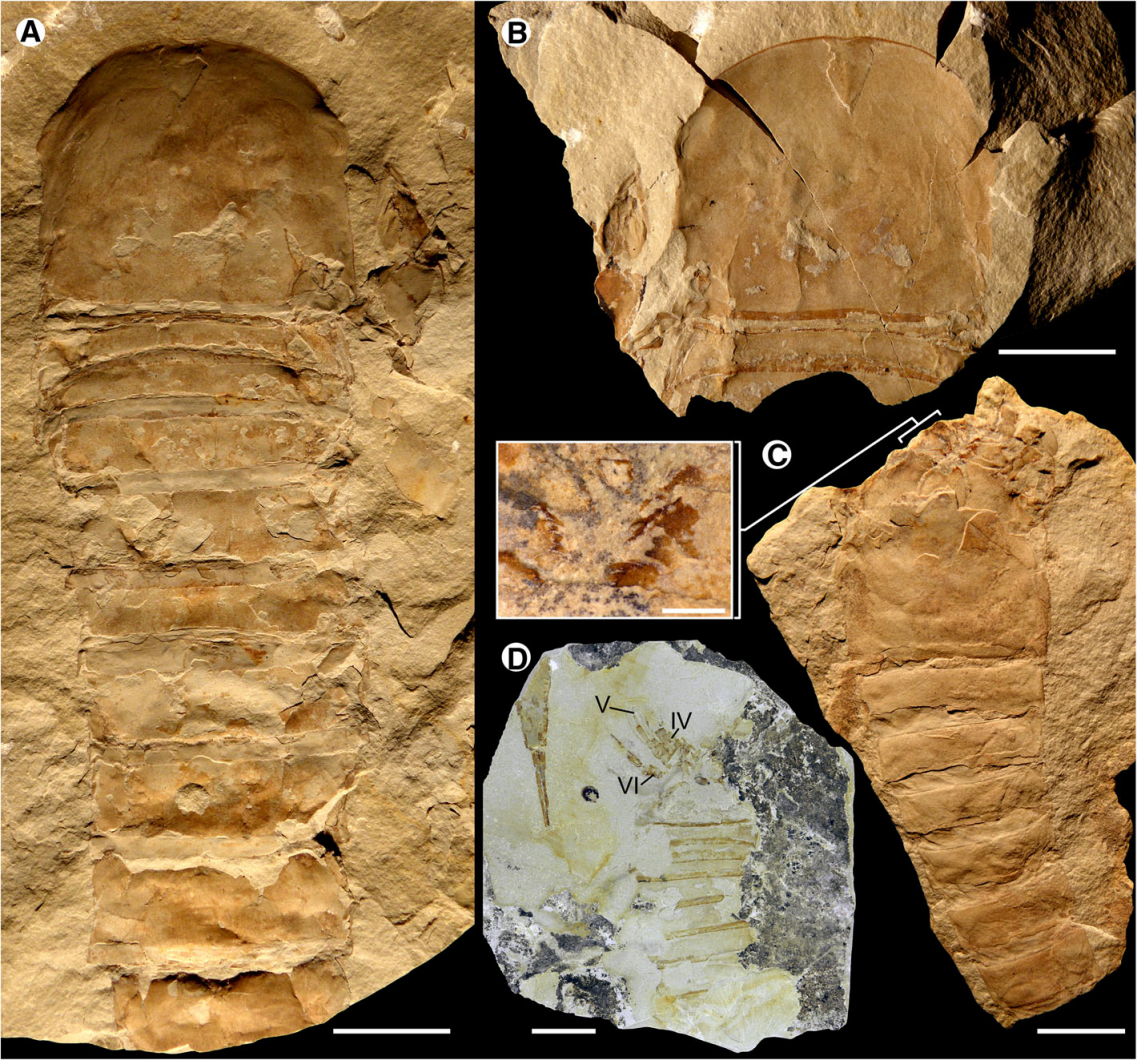
*Hoplitaspis hiawathai*, specimens preserving morphological details of the prosoma and opisthosoma. **a** UWGM 1877 (part), relatively complete specimen in dorsal view, displaying the lateral and median eyes on the prosomal shield and the dorso-lateral expansion of the second tergite. **b** UWGM 1877 (counterpart), preserving aspects of the prosomal ventral plate. **c** UWGM 2046, dorsal view of exfoliated specimen showing the coxa of the prosomal appendages in situ with the metastoma being partially overlain by the buckler ventral plate. Details of the coxal gnathobases are shown in an expanded box view. **d** UWGM 2275, two specimens preserved in association. UWGM 2275A (right) preserves details of the prosomal appendages, including a rare dorsal view of appendage VI, displaying the lateral overlap of the sixth podomere by the fifth. UWGM 2275B (left) shows details of the pretelson and telson. IV–VI = prosomal appendages IV–VI. Scale bars = 10 mm, with the exception of the expanded box of C, where the scale bar = 1 mm

**Fig. 4:**
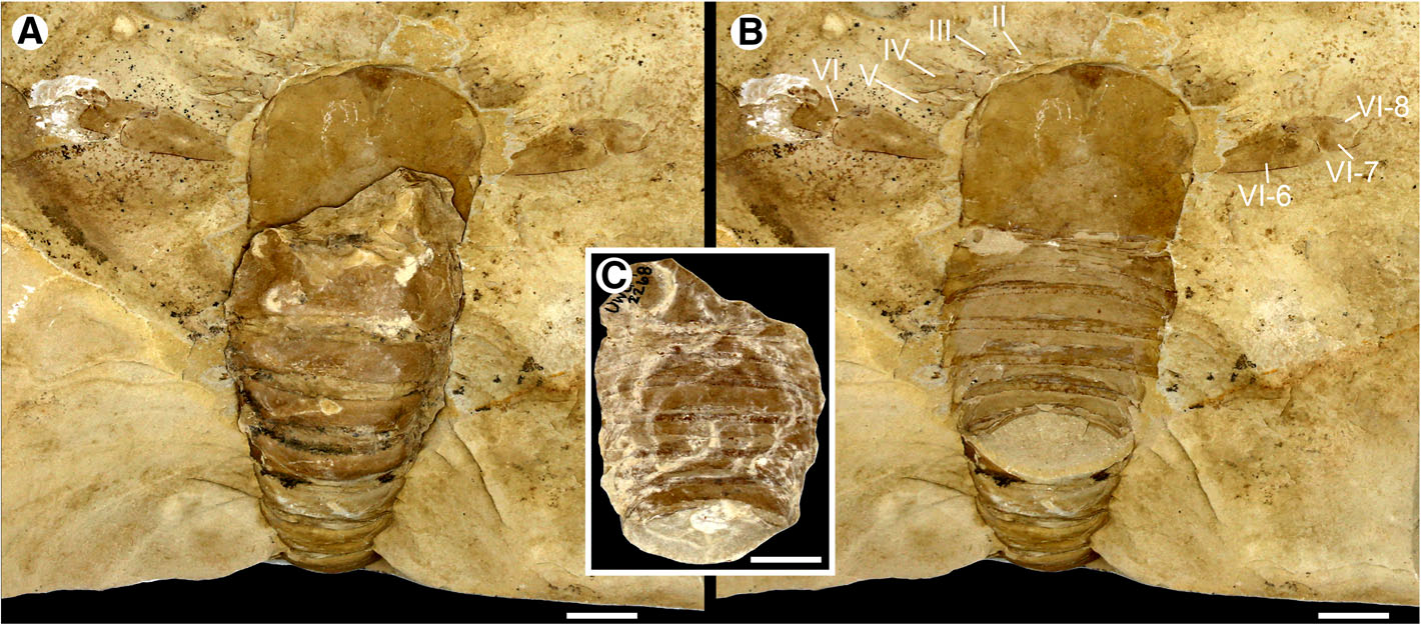
*Hoplitaspis hiawathai*, UWGM 2268 – almost complete specimen preserving prosomal appendages and buckler dorsal and ventral surface. **a** Specimen with detachable section in situ showing the buckler ventral plate. Note the projection of appendage VI from the prosoma midsection. **b** Specimen with the detachable section removed, showing the buckler tergites. Portions of the prosomal ventral plate ‘triangular area’ are preserved at the prosoma anterior. **c** Dorsal view of the detachable section, which preserves the buckler and anterior segments of the postabdomen in three-dimensional relief. II–VI = appendages II–VI, VI-6–VI-8 = appendage VI podomeres 6–8. Scale bars = 10 mm

**Fig. 5:**
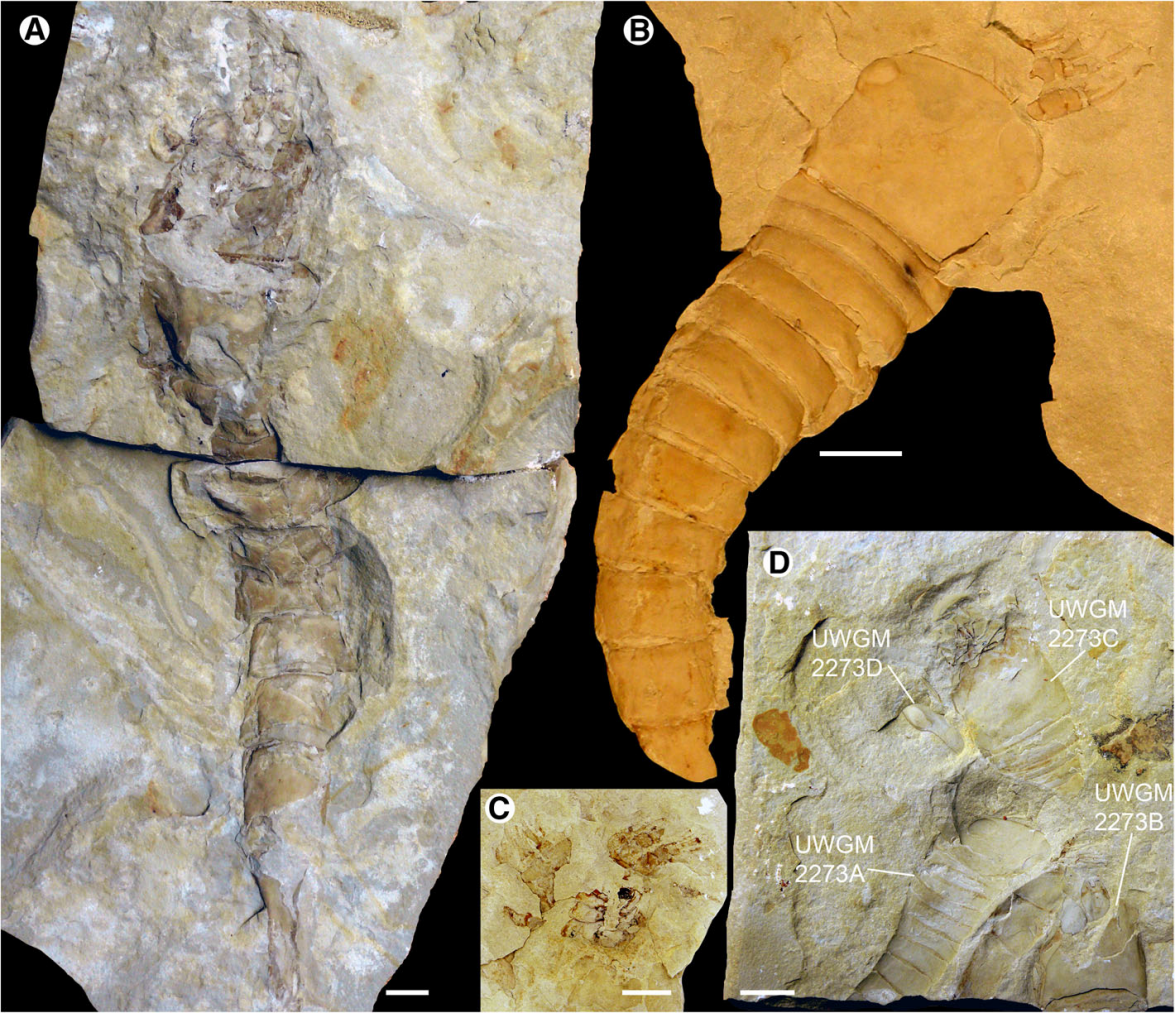
*Hoplitaspis hiawathai*, specimens preserving morphological details of the prosoma and opisthosoma. **a** UWGM 2280, large specimen preserving the full opisthosoma, including the buckler ventral plate, and robust prosomal appendage podomeres. **b** UWGM 2700, almost complete specimen preserving the lateral and median eyes, prosomal appendage armature, and opisthosoma. **c** UWGM 2276, specimen showing the prosomal appendages and gnathobases. **d** UWGM 2273, block preserving multiple specimens. UWGM 2273A displays details of the lateral eyes and prosomal appendages, UWGM 2273B affords a view of the ventral buckler plate, UWGM 2273C reveals the coxa of the prosomal appendages in situ anterior to the prosomal ventral plate, and UWGM 2273D displays a lateral view of the prosomal shield revealing the ventral expansion of the prosoma surface and the wide field of view of the lateral eye. Scale bars = 10 mm

**Fig. 6:**
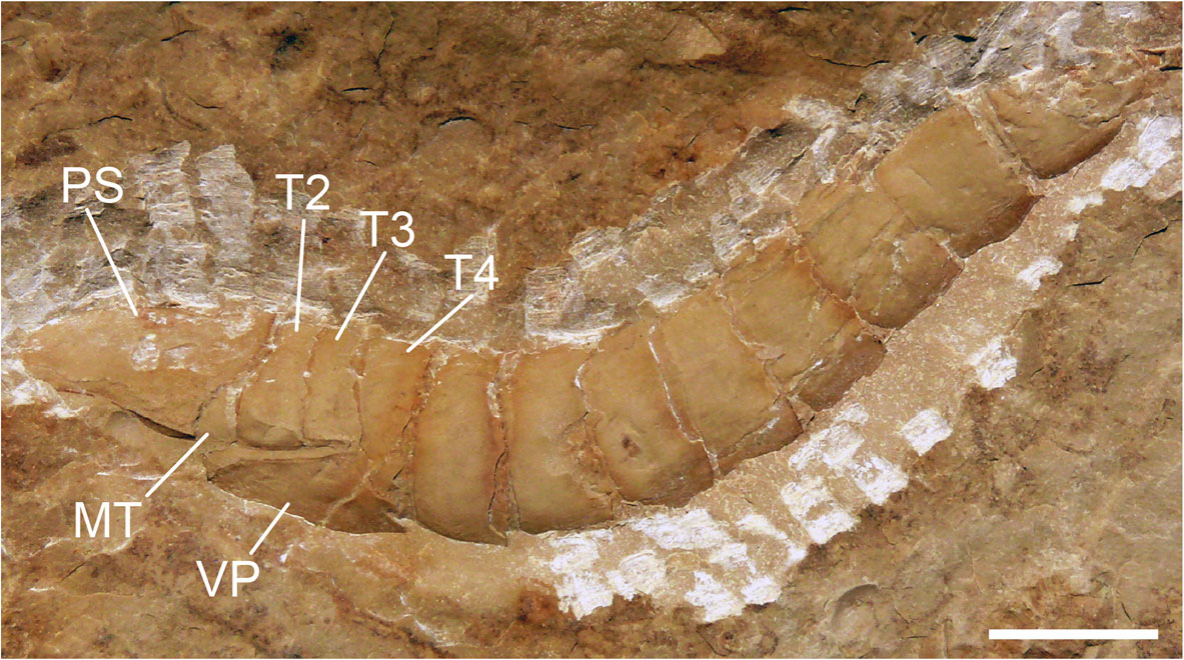
*Hoplitaspis hiawathai*, UWGM 2279A (paratype) – almost complete specimen in lateral view. The full length of the microtergite is preserved partially tucked under the prosomal shield, as is the position of the ventral plate underlying the buckler tergites. MT = microtergite, PS = prosomal shield, T2–T4 = tergites 2–4, VP = ventral plate. Scale bar = 10 mm

**Fig. 7:**
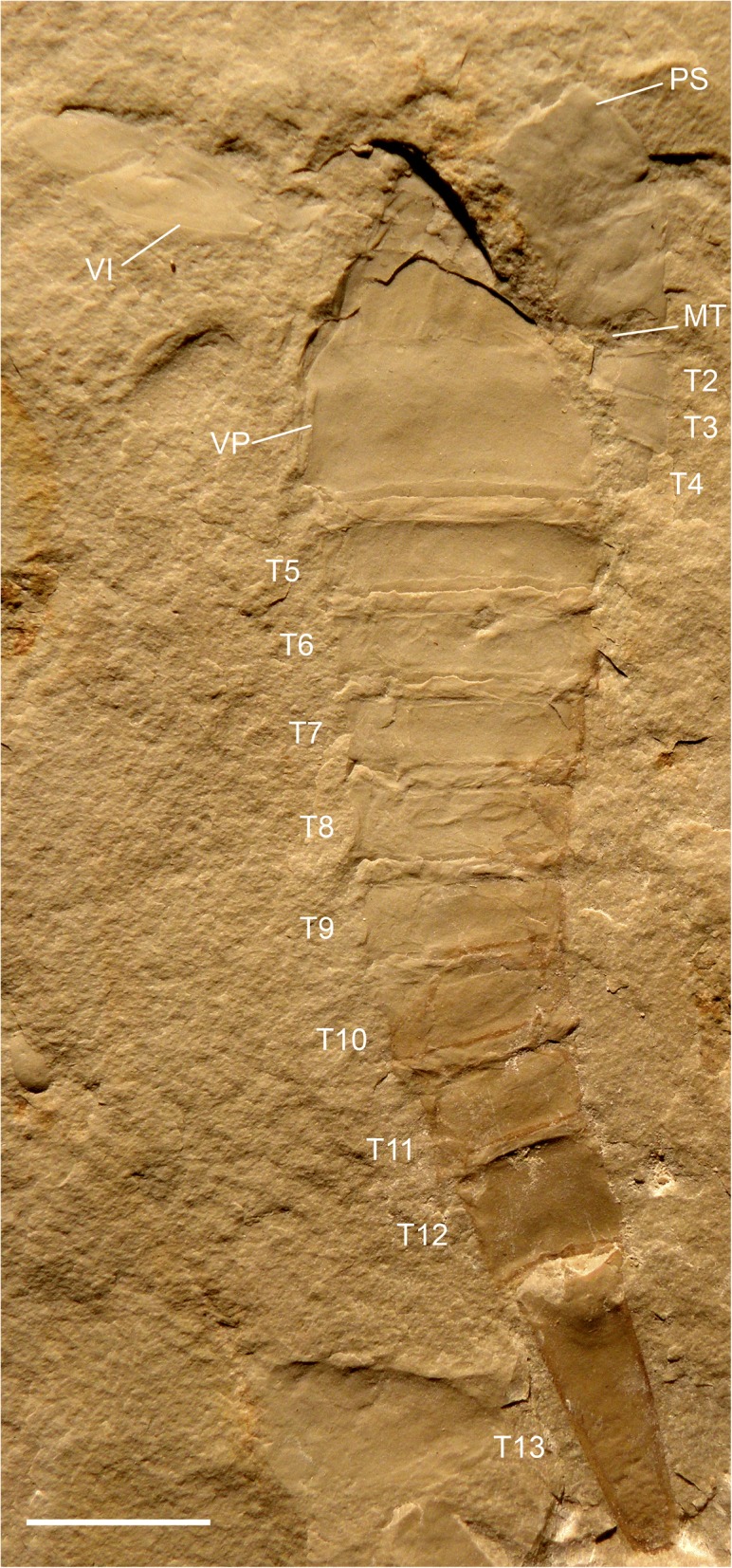
*Hoplitaspis hiawathai*, UWGM 1863 (paratype) – almost complete specimen. The specimen is split so as to reveal the dorsal prosomal shield and buckler tergites on the right, with the buckler ventral plate seen to the left. MT = microtergite, PS = prosomal shield, T2–T13 = tergites 2–13, VI = prosomal appendage VI, VP = ventral plate. Scale bar = 10 mm

**Fig. 8:**
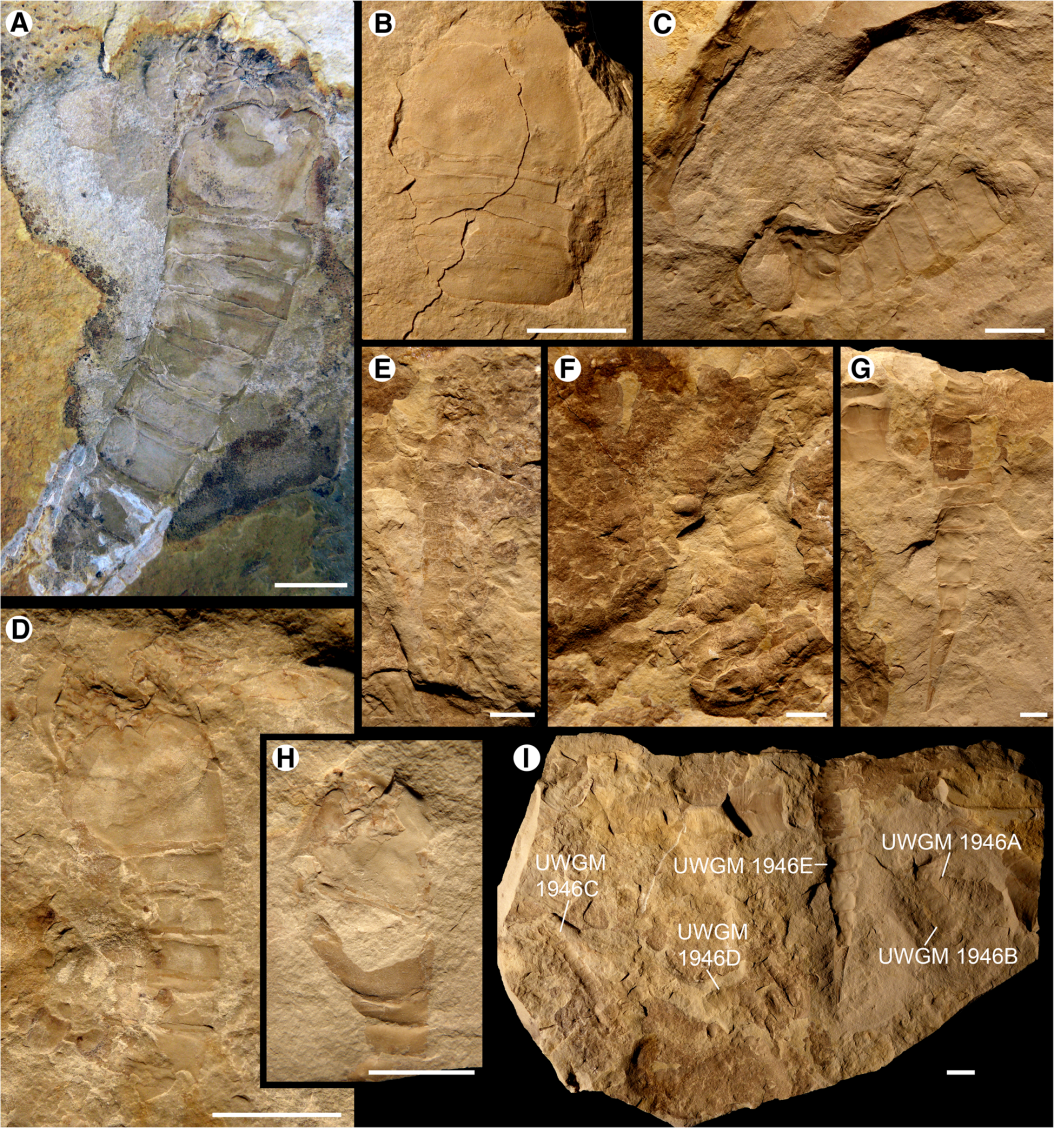
*Hoplitaspis hiawathai*, specimens preserving morphological details of the buckler and postabdomen. **a** UWGM 2284, preserving the coxae and metastoma anterior to the ventral buckler plate, which clearly shows the anterior median notch. Eight segments of the postabdomen are also preserved. **b** UWGM 2041, prosomal shield and buckler. **c** UWGM 1946A (top) and UWGM 1946B (bottom), preserving postabdominal segments. **d** UWGM 2044A, specimen showing prosomal appendage insertion with prosomal ventral plate in situ, anterior to the buckler ventral plate displaying anterior median notch. **e** UWGM 1946C, small, almost complete specimen. **f** UWGM 1946D partially disarticulated specimen exhibiting extreme curvature. **g** UWGM 1946E, postabdomen and telson. **h** UWGM 2044B, oblique view showing buckler ventral plate separating from buckler tergites. **i** UWGM 1946, block with five specimens. Scale bars = 10 mm

**Fig. 9:**
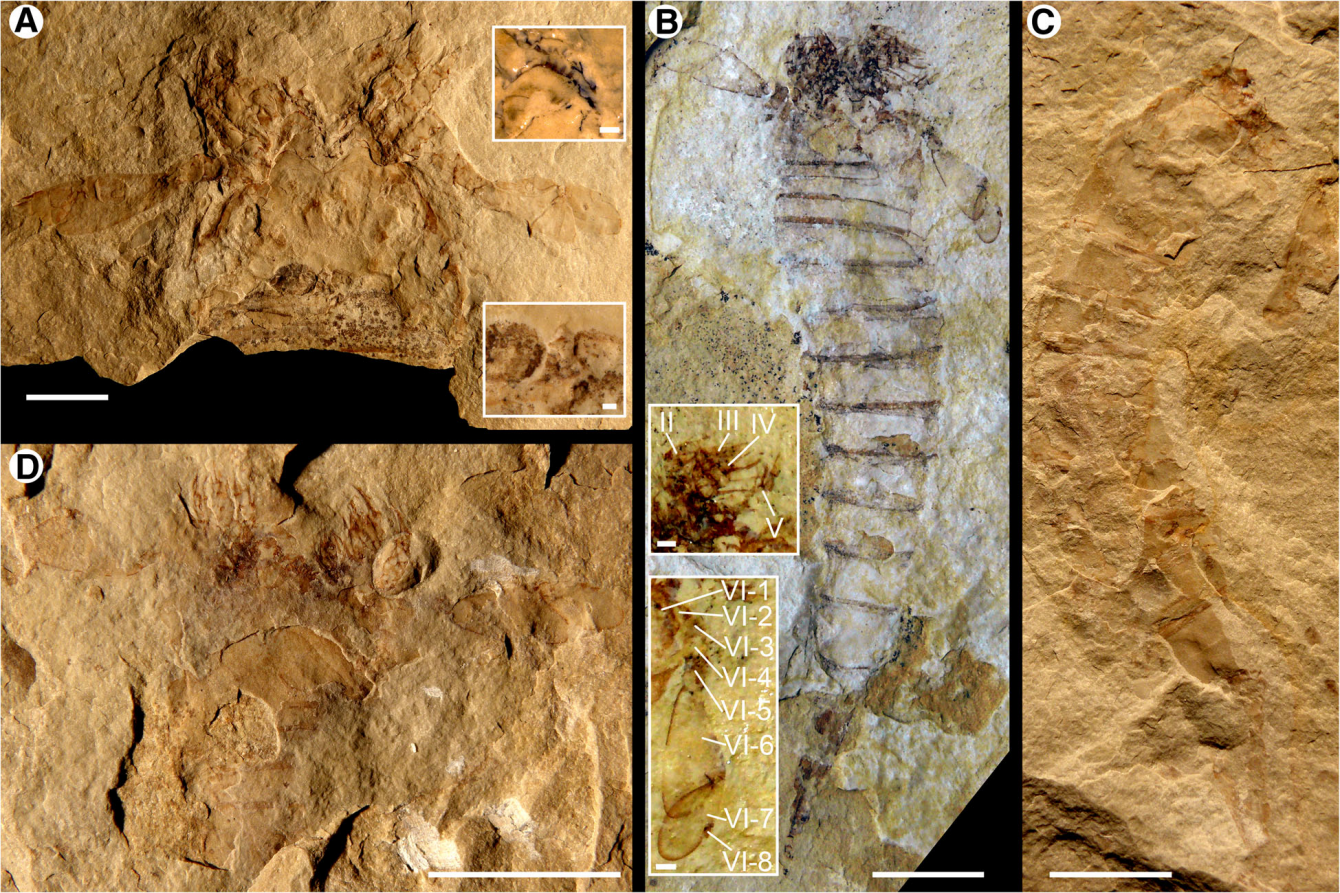
*Hoplitaspis hiawathai*, specimens preserving morphological details of the prosomal appendages and buckler. **a** UWGM 1876 (paratype), prosomal appendages and buckler. Appendages II–VI are preserved almost in their entirety, while the buckler ventral plate also preserves the posterior regions of a pair of opercula. Details of the coxae, which preserve ancillary spines along their margin, are shown in the upper expanded box while details of the gap between the opercula are shown in the lower box. **b** UWGM 2281, small specimen preserving the full opisthosoma and prosomal appendage complement, lacking only the dorsal prosomal shield. **c** UWGM 2070, opisthosoma lacking telson. **d** UWGM 1949, small specimen preserving a complete set of prosomal appendages in dorsal view lacking prosomal shield. II–V = appendages II–V, VI-1–VI-8 = appendage VI podomeres 1–8. Scale bars = 10 mm, with the exception of the expanded boxes of A and B, where the scale bars = 1 mm

**Fig. 10:**
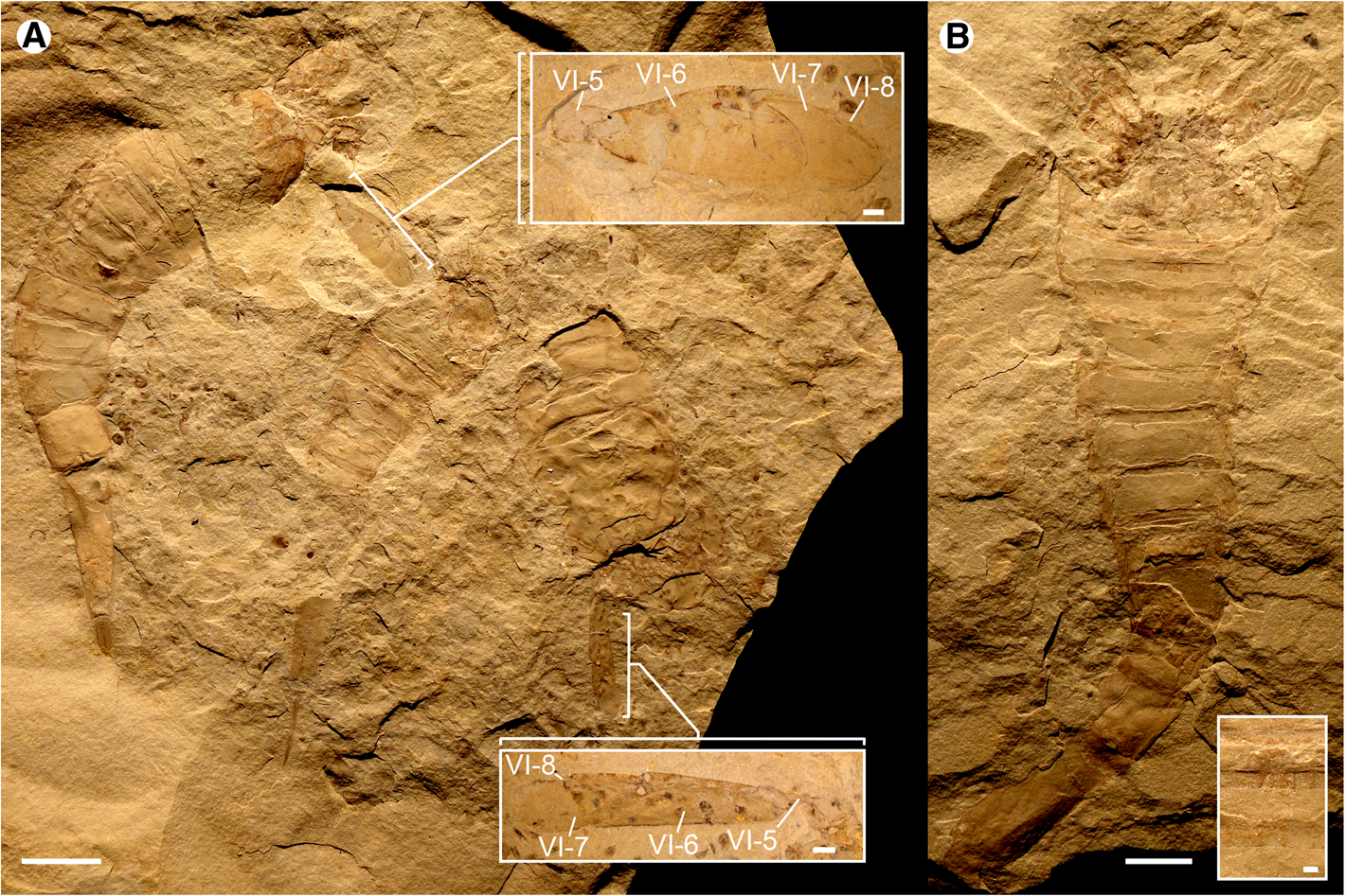
*Hoplitaspis hiawathai*, almost complete specimens preserving details of the prosomal appendages and genital appendage. **a** UWGM 1840, three specimens preserved in close association. UWGM 1840A (left) is an almost complete specimen showing the prosomal appendages, a groove down the center of the buckler that may represent the midline between opercula, and a postabdomen with a notch that may represent the anus on the twelfth segment. UWGM 1840B (center) preserves a complete opisthosoma including the telson, while UWGM 1840C (right) shows the buckler ventral plate and prosomal appendage VI. Details of the broad paddle morphology of appendage VI are shown in expanded boxes. **b** UWGM 1875 (paratype), specimen in dorsal view showing prosomal appendages and opisthosoma. The dark outline of a possible genital appendage is preserved under the buckler tergites and shown in detail in an expanded box. VI-5–VI-8 = appendage VI podomeres 5–8. Scale bars = 10 mm, with the exception of the expanded boxes, where the scale bars = 1 mm

**Fig. 11:**
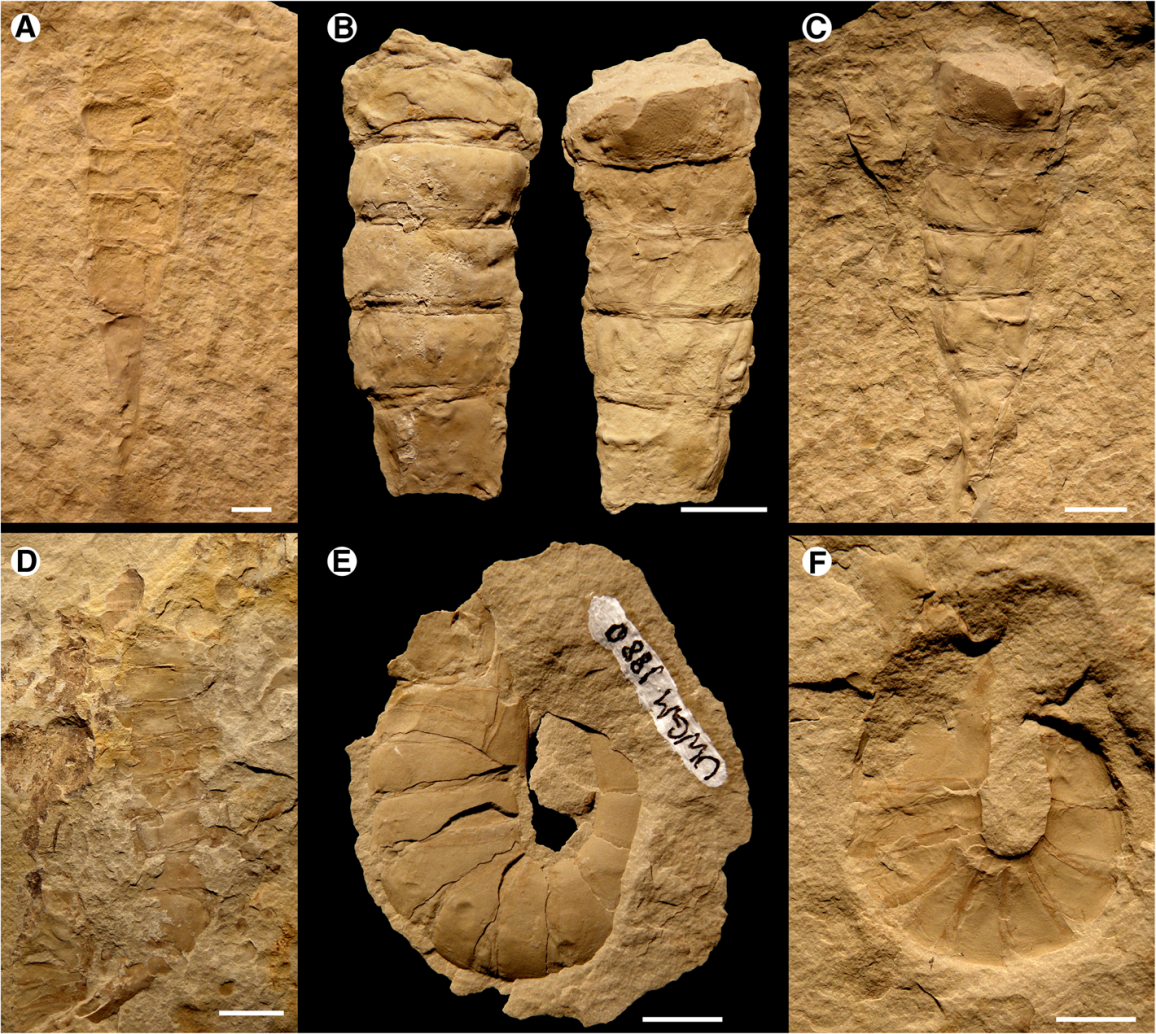
*Hoplitaspis hiawathai*, specimens preserving morphological details of the postabdomen. **a** UWGM 1838 (part), postabdomen and telson. **b** UWGM 1838 (counterpart), postabdomen preserving segments 8–12 in three-dimensional relief, from dorsal (left) and ventral (right) view. **c** UWGM 1947, postabdomen. **d** UWGM 1948, postabdomen. **e** UWGM 1880 (counterpart), specimen in oblique view showing extreme curvature of postabdominal segments and the positioning of the buckler tergites above the buckler ventral plate. **f** UWGM 1880 (part), demonstrating the extreme curvature of the postabdominal segments are due to telescoping, with the inner margins of the segments strongly overlapping while the outer margins of the segments exhibit disarticulation. Scale bars = 10 mm

**Fig. 12:**
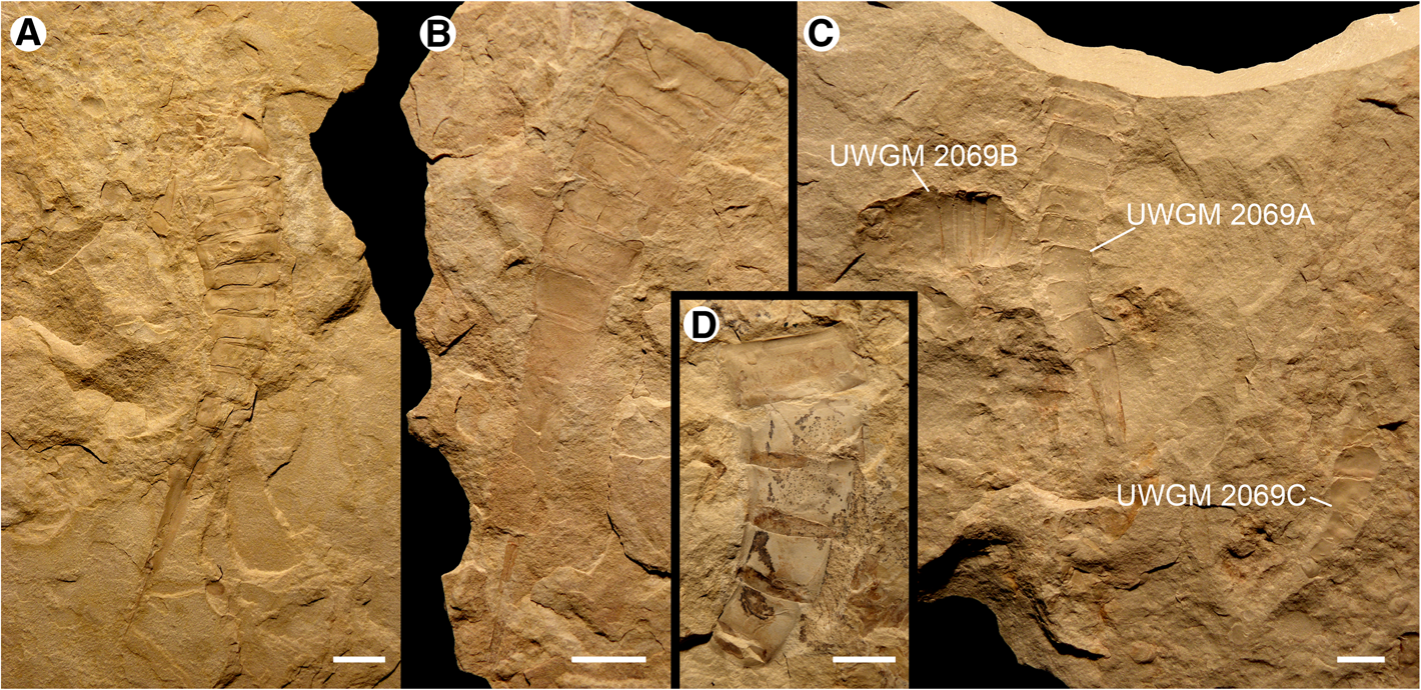
*Hoplitaspis hiawathai*, specimens preserving morphological details of the postabdomen and telson. **a** UWGM 1871 (paratype), specimen with disarticulated prosoma and buckler preserving full series of postabdominal segments and telson. **b** UWGM 2040, specimen preserving buckler posterior in dorsal view and complete postabdomen with telson. **c** UWGM 2069, block preserving multiple specimens. UWGM 2069A displays a full postabdomen including the elongate pretelson. **d** UWGM 2039, postabdominal segments 8–12. Scale bars = 10 mm

#### Etymology

Named after Hiawatha, Native American leader and co-founder of the Iroquois Confederacy, whose name is given to the Hiawatha National Forest located nearby to the Stonington Peninsula locality.

#### Material

Holotype: UWGM 1873A, large specimen preserved in dorsal and ventral three-dimensional relief, complete apart for the distal portions of the prosomal appendages, ventral buckler morphology, and the distal portions of the postabdomen and telson. Paratypes: UWGM 1863, UWGM 1871, UWGM 1875, UWGM 1876, UWGM 2279A. Additional Material: UWGM 1838, UWGM 1840, UWGM 1873B-C, UWGM 1877, UWGM 1880, UWGM 1946–1949, UWGM 2039–2041, UWGM 2044, UWGM 2046, UWGM 2063–2064, UWGM 2069–2070, UWGM 2267–2268, UWGM 2271, UWGM 2273–2276, UWGM 2279B-C, UWGM 2280–2284.

#### Horizon and locality

Upper Ordovician (Richmondian) Big Hill Lagerstätte, Big Hill Formation, Stonington Peninsula, Michigan, USA.

#### Diagnosis

As for the genus.

#### Description

The large numbers of relatively complete specimens afford an almost complete description of the external morphology of the animal. This includes rough details of the ventral appendicular structures of the buckler, which are rarely preserved in chasmataspidids.

The dorsal prosomal shield, often also referred to as the carapace, is known from 17 specimens ranging from 13 mm to 29 mm in length and 13 mm to 37 mm in width (Table 2) and comprises approximately 22% of the total body length. Length/width ratios range from 0.78 to 0.93, having an average value of 0.87. The prosomal shield lateral angles range from 87° to 93°, placing its dimensions intermediate between subquadrate and horseshoe-shaped. The lateral eyes are large, 22–28% of the prosomal shield length, ovoreniform in shape and positioned anteriorly on the prosomal shield margin intermediately between antelateral and centrilateral configuration (Figs. 2, 3a-b, 4 and 5b). The visual surface is angled anteriorly and laterally, resulting in a wide lateral field of view as indicated by UWGM 2273D (Fig. 5d) which preserves the prosomal shield in lateral aspect and demonstrates that it expands ventrally towards its anterior in order to accommodate the lateral eyes. The median ocelli are positioned centrally on the carapace and are not accommodated on an ocellar mound. The posterolateral margins of the prosomal shield are drawn out into small genal spines (Figs. 2a, 6 and 7) that are more easily visible in lateral view.

**Table 2:** *Hoplitaspis hiawathai* carapace morphology measurements

Specimen	Carapace		Lateral eye		Median ocelli	
	Length	Width	Length	Width	Length	Width
UWGM 1840B	13	15	–	–	–	–
UWGM 1863	14	10*	3	1	–	–
UWGM 1873A	29	37	8	4	1	1
UWGM 1875	23*	32*	–	–	–	–
UWGM 1877	23	26	5	2	1	1
UWGM 1880	11*	12*	–	–	–	–
UWGM 2041	15*	17*	–	–	–	–
UWGM 2044A	11	12*	–	–	–	–
UWGM 2044B	12	13	–	–	–	–
UWGM 2069B	18	18*	4	2	–	–
UWGM 2268	25	33	4	2	–	–
UWGM 2273A	11*	17*	5	3	–	–
UWGM 2273D	13*	5*	5	3	–	–
UWGM 2279A	15*	9*	–	–	–	–
UWGM 2279B	15	18	2	1	0.5	0.5
UWGM 2279C	15	16	4	1	–	–
UWGM 2700	21	24	6	3	1	1

All measurements in millimetres. Asterisk (*) indicates an incomplete measurement

The prosomal ventral plates are largely unknown, with the exception of a single specimen preserving the lateral portions of the right prosomal plate (Fig. 8d) and a median anterior structure preserved in a number of specimens in dorsal aspect (Figs. 2, 3a-b and 4). This structure appears to be somewhat resistant to compression and widens anteriorly, suggesting it is not a median suture but rather the ‘triangular area’ of Størmer [25] and Lamsdell [23]. The ventral plate is shown to narrow posteriorly (Fig. 8d), indicating they are of *Erieopterus*-type (although it is highly probable that the *Eurypterus*-type plates bearing a median suture are simply *Erieopterus*-type that have been compressed and split).

The chelicerae are not clearly preserved in the available material, but appear to be short (Fig. 5d). The post-oral prosomal appendages are frequently preserved. All prosomal appendages are directed anteriorly (Figs. 3d, 5c, 9a, d and 10b), although this could be exaggerated through anterior displacement during the molting process. Appendages II–V increase in size consecutively (Tables 3 and 4) and are largely uniform, with only appendage II differentiated through having seven podomeres in total while appendages III–V have eight. These appendages are exceedingly robust (Fig. 5a, b), comprising a series of podomeres with enlarged raptorial armature (Figs. 5c, 9b, d and 10b). The limbs are inserted on the underside of the prosoma with the coxae arrayed in parallel to one another (Figs. 2b, 3c, 8a and 9a, b, d) forming a gnathobasic battery leading to the oral opening as in horseshoe crabs [26–29], eurypterids [19, 23, 30], and other chasmataspidids [12]. The coxae are elongated and narrow (Figs. 5d and 8a), with upward-curving gnathobasic spines (Figs. 2b and 3c). A row of short, robust ancillary spines is present lining the ventral surface of the coxae on UWGM 1876 (Fig. 9a), with approximately six spines present on each coxa.

**Table 3:** *Hoplitaspis hiawathai* prosomal appendage II & III measurements

Specimen	Appendage	Podomeres							
		Coxa	2	3	4	5	6	7	8
UWGM 1840A	III	2/1	2/1	−/−	−/−	−/−	−/−	−/−	−/−
UWGM 1840B	II	−/−	−/−	−/−	1/2	1/1	1/1	−/−	–
	III	−/−	−/−	−/−	2/2	1/1	1/1	−/−	−/−
UWGM 1873A	II	5/2	4/2	3/2	3/2	2/2	−/−	−/−	–
	III	7/3	4/3	4/2	4/2	2/2	2/2	3/2	2/1
UWGM 1875	II	−/−	−/−	−/−	3/3	3/3	2/3	6/1	–
	III	−/−	−/−	3/2*	3/3	2/2	2/1	3/1	3/1
UWGM 1876	II	4/2	3/2	3/2	−/−	−/−	−/−	−/−	–
	III	5/2*	2/3	2/2	2/2	2/2	1/2	−/−	−/−
UWGM 1949	II	−/−	−/−	−/−	−/−	1/1	1/1	1/0.5	–
	III	−/−	−/−	−/−	−/−	−/−	1/1	2/1	2/0.5
UWGM 2044A	II	2/1	1/1	1/1	1/1	1/1	−/−	−/−	–
	III	2/1	1/1	−/−	−/−	−/−	−/−	−/−	−/−
UWGM 2046	III	3/1	2/2	2/2	−/−	−/−	−/−	−/−	−/−
UWGM 2063A	II	3/1	2/1	−/−	−/−	−/−	−/−	−/−	–
	III	3/1	2/1	−/−	−/−	−/−	−/−	−/−	−/−
UWGM 2268	II	−/−	−/−	−/−	2/2	2/2	2/2	2*/1	–
	III	−/−	−/−	−/−	1*/2	2/2	1/2	2*/1	−/−
UWGM 2273A	III	−/−	−/−	−/−	−/−	1/1	1/1	1/1	2/0.5
UWGM 2273C	II	2/1	−/−	−/−	−/−	−/−	−/−	−/−	–
	III	3/1	2/1	−/−	−/−	−/−	−/−	−/−	−/−
UWGM 2276	III	4*/1*	−/−	−/−	4/3	2/2	4/2	3/1	−/−
UWGM 2281	II	−/−	−/−	1/1	1/1	1/1	1/0.5	−/−	–
	III	−/−	−/−	−/−	1/1	1/1	1/1	1/0.5	−/−
UWGM 2700	II	−/−	−/−	−/−	−/−	−/−	1*/2	5/1	–
	III	−/−	−/−	−/−	−/−	3*/3	2/3	3/1	7/1

Length/width. All measurements in millimetres. Asterisk (*) indicates an incomplete measurement

**Table 4:** *Hoplitaspis hiawathai* prosomal appendage IV & V measurements

Specimen	Appendage	Podomeres							
		Coxa	2	3	4	5	6	7	8
UWGM 1840A	IV	3/1*	2/1*	1/2	2/1	−/−	−/−	−/−	−/−
	V	4/2	−/−	2/1*	2/1	−/−	−/−	−/−	−/−
UWGM 1840B	IV	−/−	−/−	−/−	2/2	1/1	−/−	−/−	−/−
UWGM 1873A	IV	7/3	3*/3	4/3	3/3	−/−	−/−	−/−	−/−
	V	7/4	−/−	3*/3	3/2*	−/−	−/−	−/−	−/−
UWGM 1875	IV	−/−	−/−	3/3	3/3	2/3	1/1	3/1	3/1
	V	−/−	−/−	3/3	3/3	2/3	1/2	4/1	4/1
UWGM 1876	IV	6/3*	2/4	3/3	3/3	3/2	1/2	3/1	2*/1
	V	7/3	3/2	3/2	2/2	2/2	−/−	−/−	−/−
UWGM 1949	IV	−/−	−/−	−/−	−/−	−/−	1/1	2/1	2/0.5
	V	−/−	−/−	−/−	−/−	1/1	1/1	1/0.5	1/0.5
UWGM 2044A	IV	2/1	1/1	1/1	−/−	−/−	−/−	−/−	−/−
	V	2/1	1/1	1/1	−/−	−/−	−/−	−/−	−/−
UWGM 2046	IV	5/2	3/2	2/2	−/−	−/−	−/−	−/−	−/−
	V	6/1*	3/2*	2/2	1*/2	−/−	−/−	−/−	−/−
UWGM 2063A	IV	4/2	2/2	−/−	−/−	−/−	−/−	−/−	−/−
	V	4/2	−/−	−/−	−/−	−/−	−/−	−/−	−/−
UWGM 2268	IV	−/−	−/−	−/−	−/−	3/3	2/3	5/1	4*/1
	V	−/−	−/−	−/−	−/−	1*/3	2/3	5/1	2*/1
UWGM 2273A	IV	−/−	−/−	−/−	−/−	−/−	−/−	3/1	4/1
UWGM 2273C	IV	4/3	2/2	−/−	−/−	−/−	−/−	−/−	−/−
	V	5/2	−/−	−/−	−/−	−/−	−/−	−/−	−/−
UWGM 2275A	IV	−/−	−/−	−/−	2/2	2/2	1/2	−/−	−/−
	V	−/−	−/−	−/−	2/2	2/2	1/2	3/1	3/1
UWGM 2276	IV	6*/3	−/−	−/−	4/4	3/3	2/3	1*/1*	−/−
	V	7/3	−/−	−/−	5/4	3/3	2/3	4/1	1*/1
UWGM 2280	IV	−/−	−/−	−/−	7/6*	6/4*	4/3*	3*/1*	−/−
	V	−/−	−/−	10/8	6/6	6/5	4/5	−/−	−/−
UWGM 2281	IV	−/−	−/−	1/1	1/1	1/1	0.5/1	1/0.5	1/0.5
	V	−/−	1/1	1/1	1/1	1/1	0.5/0.5	1/0.5	−/−
UWGM 2284	IV	3/2	1/2	−/−	−/−	−/−	−/−	−/−	−/−
	V	5/2	2/2	−/−	−/−	−/−	−/−	−/−	−/−
UWGM 2700	IV	−/−	−/−	−/−	2*/2*	3/3	2/3	3/1	6/1

Length/width. All measurements in millimetres. Asterisk (*) indicates an incomplete measurement

The second podomere on prosomal appendages II–V is devoid of armature (Figs. 2b, 3c and 8a). The third to sixth podomeres each bear a pair of thickened, elongate spines that insert into sockets positioned laterally on the distal margin of each podomere (Figs. 5a, b, c and 9a, b). The spines extend beyond the succeeding podomere to the midpoint of the podomere after that, with the exception of the last pair of spines (occurring on the sixth podomere) that are enlarged in comparison to the preceding spine pairs and extend to the tip of the terminal spinous podomere (Figs. 3d, 5b, C and 9a). The penultimate (seventh) podomere of appendages III–V is elongated and lacks armature (Figs. 3d, 5c and 9a, d), with the preceding podomere (podomere six) being shortened and bearing the larger final pair of spines (Figs. 3d, 5b, c and 9a). In appendage II, where the sixth podomere is the penultimate podomere, the podomeres are undifferentiated and bear uniform armature until the spinous terminal podomere (Fig. 9d).

Appendage VI is larger than the preceding appendages and comprises eight highly differentiated podomeres (Table 5) that are modified into a swimming paddle (Figs. 4, 7, 9a, b, d and 10a). As in eurypterids [22], the paddle appears to have consistently rotated about the second or third podomere during compression, resulting in the dorsal margin of the distal podomeres oriented so as to face anteriorly in the fossil specimens. The coxa is enlarged (Figs. 2b, 3c and 8a), being almost as wide as long, with no anterior ‘ear’ expansion as occurs in Eurypterina [23, 31]. Despite the size of the coxa, the gnathobase is not well developed and is approximately equal in size to the gnathobases of the preceding appendages (Figs. 3c and 8a). The second podomere inserts anteriorly on the coxa (Fig. 3c) resulting in an extreme anterior deflection of the limb that is counteracted by a consistent angle produced at the joint between the third and fourth podomeres (Fig. 9a). This results in appendage VI projecting from underneath the prosomal shield around the vertical midline (Figs. 4, 7 and 9b). The resulting 90° angle between the paddle podomeres and the prosomal shield margin is consistent across the majority of specimens (Figs. 4, 7, 9a, d and 10a) indicating that this is the resting position for the limb, while other specimens show that the paddle had at least a 90° field of movement stemming from the articulations of the fourth, fifth and sixth podomeres (Fig. 9b). The fifth podomere is highly modified, changing from the normal tubular podomere cross section proximally to being longitudinally flattened distally (Fig. 5c). The distal podomere margins are extended into triangular projections (Figs. 7, 9a and 10a) that laterally overlap the succeeding sixth podomere (Fig. 3d), permitting a large degree of dorso-ventral articulation but blocking any lateral movement. Podomere six is elongate and expands distally, with a lobate ventrodistal expansion that increases the surface area of the paddle (Fig. 9a, d). Podomere seven is also expanded ventrally, giving it an oval outline, and articulates with the sixth podomere through a dorsal pivot joint (Figs. 4, 7). This results in podomere seven being able to extend out to increase the paddle surface area (Fig. 9b) or fold into the ventrodistal lobe of podomere six (Fig. 10a). The small and spinous terminal podomere (podomere eight) is located midway along podomere seven and inserts into a notch on the podomere dorsal margin, resulting in the paddle technically having a chelate configuration (Figs. 4, 7, 9a, b and 10a).

**Table 5:** *Hoplitaspis hiawathai* prosomal appendage VI measurements

Specimen	Podomeres							
	Coxa	2	3	4	5	6	7	8
UWGM 1840A	4/2	1/2	−/−	−/−	3/2	11/5	7/4	1/0.5
UWGM 1840C	5/6	1/2	3/2	2/2	−/−	12/3	5*/3	−/−
UWGM 1863	−/−	−/−	−/−	2/1	2/2	10/4	7/3	1/0.5
UWGM 1871	−/−	−/−	−/−	−/−	2/1	10/3	4/3	−/−
UWGM 1873A	12/12	2/5	−/−	−/−	−/−	−/−	−/−	−/−
UWGM 1875	7/8	2/4	2/4	2/3	2*/3	−/−	−/−	−/−
UWGM 1876	−/−	−/−	3/3	3/3	5/3	15/6	8/5	2/1
UWGM 1949	−/−	−/−	−/−	−/−	−/−	6*/3	4/2	1/0.5
UWGM 2044A	1*/4	1/1	1/1	2/1	1/1	6/2	4*/2	1/0.5
UWGM 2046	7*/11	1/3	1/2	−/−	−/−	−/−	−/−	−/−
UWGM 2063A	5/6	1/2	1/2	−/−	−/−	−/−	−/−	−/−
UWGM 2268	−/−	−/−	−/−	−/−	−/−	17*/6	11/5	2/1
UWGM 2275A	−/−	1/1*	2/1*	3/1*	3/1*	5*/1*	−/−	−/−
UWGM 2276	9/3*	−/−	3/2	3/2	2*/1*	−/−	−/−	−/−
UWGM 2281	−/−	1/1	2/1	1/1	2/1	7/2	5/2	1/0.5
UWGM 2284	10/3*	1/2	−/−	−/−	−/−	−/−	−/−	−/−

Length/width. All measurements in millimetres. Asterisk (*) indicates an incomplete measurement

The metastoma is preserved in four specimens and is obovate in shape (Fig. 2b), with the widest point occurring in the anterior third. The anterior margin has a shallow notch with rounded shoulders (Figs. 3c, 5c and 8a), with the metastoma cuticle within the notch dark and thickened indicating a role in mastication. Although the metastoma posterior is not preserved, the lateral margins begin to converge considerably after the greatest width is attained (Fig. 2b).

The opisthosoma comprises 13 segments and is divided into an anterior preabdomen and posterior postabdomen. The preabdomen comprises four tergites (Table 6) which together make up 13% of the total body length. The first tergite is shorter than the succeeding tergites, being about three quarters the length of the other buckler tergites and partially covered dorsally by the posterior margin of the prosomal shield (Figs. 8b and 9b), and is homologous to the microtergite of other diploaspidids. This shortened first tergite is freely articulating between the prosoma and the fused buckler tergites (Fig. 6) and narrows laterally, with the first fused tergite of the buckler (the second opisthosomal tergite) exhibiting a corresponding anterior lateral expansion (Figs. 2a and 3b), similar to the ‘shoulders’ seen in other diploaspidids [11] but lacking the dorsal inflection. The second and third tergites are moderately curved while the posterior margin of the fourth tergite is flattened, corresponding to the posterior of the buckler (Figs. 2a, 3a and 5b). These tergites are largely devoid of ornamentation, with well-preserved specimens showing that each tergite has a row of elongate scales running across the posterior margin (Figs. 3b and 4).

**Table 6:** *Hoplitaspis hiawathai* preabdomen measurements

Specimen	1	2	3	4	Ventral plate
UWGM 1840A	–	–	–	4/18	17/17
UWGM 1840B	–	3/16	3/17	3/15	11/17
UWGM 1840C	–	–	–	4/22	17/22
UWGM 1863	1*/4*	2/20	2/20	3/19	10/20
UWGM 1871	1/17	2/19	2/19	2/17	10/19
UWGM 1873A	2*/33	5/36	5/35	5/33	21*/34
UWGM 1873B	1*/6*	2/8*	2/8*	3/9*	11/4*
UWGM 1873C	1*/17*	3/19*	3/18*	4/18*	7*/2*
UWGM 1875	3/29	4/32	5/32	5/28	–
UWGM 1876	–	–	–	–	20/26
UWGM 1877	1*/25	4/27	5/27	6/27	–
UWGM 1880	1*/5*	4/13*	4/14*	6/17*	15/5*
UWGM 1946B	–	3/10*	3/11*	4/12*	16/11*
UWGM 1946E	–	4/15*	4/15*	5/21*	–
UWGM 1949	–	–	1*/7*	2/8*	7/10
UWGM 2040	–	–	2*/9*	4/19	–
UWGM 2041	2/18*	3/17*	3/16*	3/16*	–
UWGM 2044A	–	–	–	–	11/12
UWGM 2044B	–	–	–	–	10/13
UWGM 2046	–	4/2*	4/2*	5/1*	19/24
UWGM 2063A	–	–	–	–	10/14
UWGM 2063C	–	–	–	–	8/9
UWGM 2063D	–	–	–	–	10/11*
UWGM 2069B	1*/15	3/15	3/15	3/15	–
UWGM 2070	–	–	–	3/11	11/11
UWGM 2268	2/28	3/32	4/33	5/32	14/33
UWGM 2273A	–	2/15*	3/14*	3/14*	–
UWGM 2273B	–	–	–	–	18/21
UWGM 2273C	–	–	–	–	19/19
UWGM 2275A	–	3/14*	3/13*	3/17*	–
UWGM 2279A	3/8*	4/8*	4/7*	4/7*	12/5*
UWGM 2279B	1*/17	2/18	3/18	3/17	–
UWGM 2279C	–	2/14	2/15	2/14	–
UWGM 2280	–	–	–	–	25*/32
UWGM 2281	1/9*	2/12*	2/12*	3/13*	8/14
UWGM 2284	–	–	–	–	17/23
UWGM 2700	2/20*	3/24	4/24	4/22	–

Length/width. All measurements in millimetres. Isolated tergites were assigned to a segment based on size and differences in ornamentation. Asterisk (*) indicates an incomplete measurement

The fusion of tergites 2–4 is difficult to discern from complete dorsally-preserved specimens (Figs. 2, 3a, 5b, 9b and 10b). Numerous specimens, however, preserve the ventral buckler plate (Figs. 3c, 5a, d, 8a, c and 9c). This plate is rectangular with a deep anterior notch (Figs. 8d and 9a) and comprises the fused sternites of segments 2–4. The ventral plate does not directly underlie the tergites, as shown by a number of three-dimensional specimens preserved in dorsal and ventral aspect (Figs. 2 and 4) and one specimen (Fig. 7) that preserves the ventral plate and the lateral portions of the buckler tergites. That the ventral buckler plate is composed of the sternites is demonstrated unequivocally by a number of specimens preserved in lateral aspect (Figs. 2, 6, 8h and 11e, f).

Little is preserved of the abdominal appendages, although UWGM 1840A (Fig. 10a) exhibits a groove running down the center of the ventral buckler that may represent the medial suture of the opercula. Portions of the posterior pair of opercula are preserved in UWGM 1876 (Fig. 9a), showing them to be unfused medially. The genital appendage is preserved in a single specimen, UWGM 1875 (Fig. 10b), as a dark organic stain overlain by the buckler tergites. The appendage is short, measuring 8 mm long and 3 mm wide, and extends to the level of the posterior of the third tergite. The genital appendage consists of two segments, with the second segment bearing paired ridges that run along its length.

The postabdomen consists of nine freely articulating segments that consecutively decrease in width (Table 7). The postabdomen comprises 53% of the total body length (Figs. 6, 7, 8e, f, g, i and 9c). The postabdominal segments are ankylosed rings of laterally fused tergite/sternite pairs (Fig. 11b, c, e, f) that transition from the uniform segments 5–10 to the progressively more elongated segments 11–13 (Figs. 11a, d and 12a, b, c, d). The pretelson is unusually elongated compared to the preceding segments, being over twice the length of segment 12 (Figs. 7, 8g, 9c, 10a, b, 11a and 12a, b, c). Interestingly, one specimen (UWGM 1840A (Fig. 10a)) has a medial posterior notch on segment 12 that could represent the anus. If this is the case, then the pretelson is post-anal, which would suggest that it is not homologous to the pre-anal pretelson of eurypterids and scorpions. The telson is lanceolate, with a dorsal carina (Fig. 3d). The telson is exceedingly narrow, making it almost needle-like (Figs. 10 and 12a, b).

**Table 7:** *Hoplitaspis hiawathai* post-buckler tergite and telson measurements

Specimen	4	6	7	8	9	10	11	12	13	Telson
UWGM 1838A	–	–	–	10/25	10/24	12/24	12/23	17/19	34/11	4*/2*
UWGM 1838B	–	–	3*/17*	8/23	9/21	9/20	10/19	12/15	–	–
UWGM 1840A	5/19	5/19	5/17	5/15	6/15	6/13	6/12	8/8	21/7	5*/2
UWGM 1840B	3/15	3/14	3/14	4/12	4/11	2*/10	–	–	13*/5	12/1
UWGM 1840C	5/21	5/18	4*/10*	–	–	–	–	–	–	–
UWGM 1863	5/16	5/14	5/13	5/12	5/11	5/10	6/9	6/8	17/6	–
UWGM 1871	5/16	5/15	5/14	6/12	6/10	6/8*	7/6*	9/6	21/5	15/2
UWGM 1873A	7/31	7/27	8/25	8/22	8/21	9/19	10/16	8*/9*	–	–
UWGM 1873B	4/11*	4/10*	4/10*	4/10*	4/8*	4/8*	5/7*	–	–	–
UWGM 1873C	5/19	6/18	7/16	5/15	5/12*	5/11*	6*/10	–	–	–
UWGM 1875	7/26	6/24	7/22	7/20	7/17	8/15	9/12	12/12	30/9	–
UWGM 1877	7/24	7/24	8/24	8/22	8/22	7/20	–	–	–	–
UWGM 1880	6/16	6/16	6/15	6/14	6/12	6/12	7/10	9/8	–	–
UWGM 1946A	4/13	4/12	4/12	4/10	4/9	–	–	–	–	–
UWGM 1946B	6/13*	6/11*	6/12*	6/12	6/11	–	–	–	–	–
UWGM 1946C	4/11	4/8*	4/8	4/7	4/7	4/7	4/6	6/6	11*/3	–
UWGM 1946D	5/15	5/12*	4/11*	49/34*	4/12	5/11	5/11	–	–	–
UWGM 1946E	10/25	9/25	9/22	8/21	9/19	11/17	10/14	10/11	26/8	12*/3
UWGM 1947	–	–	–	7/21	7/20	9/20	10/18	12/14	17*/9	–
UWGM 1948	5/15*	5/16	5/15*	5/17	5/14*	6/15	6/9*	9/8*	16/4	–
UWGM 1949	2/6*	2/3*	–	–	–	–	–	–	–	–
UWGM 2039	–	–	–	10/22	11/20	13/17	13/14	12/11	–	–
UWGM 2040	4/18	4/17	5/16	5/15	5/14	6/13	6/10	9/9	21/7	20/2
UWGM 2041	4/14*	–	–	–	–	–	–	–	–	–
UWGM 2044A	3/8*	3/5*	3/6*	3/4*	3/4*	3/4*	–	–	–	–
UWGM 2044B	3/7*	3/6*	3/5*	–	–	–	–	–	–	–
UWGM 2046	7/21	7/19	7/17	7/15	7/14	7/13	–	–		–
UWGM 2063A	4/12	4/12	4/8*	4/7*	2*/4*	–	–	–		–
UWGM 2063B	3/10*	3/10*	4/11	4/11	4/10	4/8	6/7	6/6	13/4	6/2
UWGM 2063C	5/9	5/9	4/8	3/7	2*/5*	3/6	3/6	5/5	10/3	–
UWGM 2063D	5/10	4/9	5/9	5/7*	5/10	4/8*	6/9	7/7	13*/6	–
UWGM 2069A	6/18	6/16	7/15	6/13	6/12	7/11	7/9	8/7	21/7	–
UWGM 2069B	4/15	3/12*	3/8*	–	–	–	–	–	–	–
UWGM 2069C	4*/8*	7/9*	7/10*	8/7*	5*/4*	–	–	–	–	–
UWGM 2070	4/9*	4/10*	4/9*	3/11*	3/11*	3/11*	4/8*	6/4*	13*/6	–
UWGM 2268	6/32	6/27*	6/23*	6/22*	6/18*	6/15*	–	–	–	–
UWGM 2273A	3/14	3/13	4/12	4/11	5/11	5/8*	3*/3*	–	–	–
UWGM 2273B	3/20	–	–	–	–	–	–	–	–	–
UWGM 2273C	4/17	4/14*	3/9*	4/6*	–	–	–	–	–	–
UWGM 2275A	4/16*	4/17*	4/16*	4/8*	–	–	–	–	–	–
UWGM 2275B	–	–	–	–	–	–	–	–	14*/5*	12/2
UWGM 2279A	5/12	5/11	6/11	6/11	6/10	6/10	7/9	7/7	5*/3*	–
UWGM 2279B	6/16	6/15	6/14	6/12	6/10	6/9	6/8	5*/5*		–
UWGM 2279C	2*/14	3/13	2/13	2/10*	3/10	–	–	–	–	–
UWGM 2280	14/25*	13/17*	11*/29	12/25*	10*/26	15/24*	16/25	23/21	40*/14	–
UWGM 2281	4/13	4/12	4/11	4/10	4/9	4/8	4/7	6/6	11/4	7/2
UWGM 2284	5/20	5/18	5/17	5/15	5/14	6/12	7/12	8/10	3*/7*	–
UWGM 2700	5/21	5/19	6/18	6/18	7/17	7/16	8/14	10/13	8*/9	–

Length/width. All measurements in millimetres. Isolated tergites were assigned to a segment based on size and differences in ornamentation. Asterisk (*) indicates an incomplete measurement

#### Remarks

The chasmataspidid identity of *Hoplitaspis* is demonstrated unequivocally by its possession of a fused anterior buckler and 13 opisthosomal segments. *Hoplitaspis* is therefore the most completely known chasmataspidid and only the second chasmataspidid species known from the Ordovician. The Big Hill specimens described herein exhibit some similarity to material (e.g. specimens MM I-4036B, I-4060, I-4308, I-4582) from the roughly contemporaneous William Lake and Airport Cove Lagerstätten of Manitoba, Canada [32, 33]. While the Manitoba material has been described as representing eurypterids, specimens MM I-4036B and MM I-4308 may possess a buckler and MM I-4060 exhibits a similar dorsal prosomal shield morphology to that of *Hoplitaspis*, while the appendages appear to bear a similar armature to the Big Hill species*.*

## Discussion

### Mode of life

**Fig. 13:**
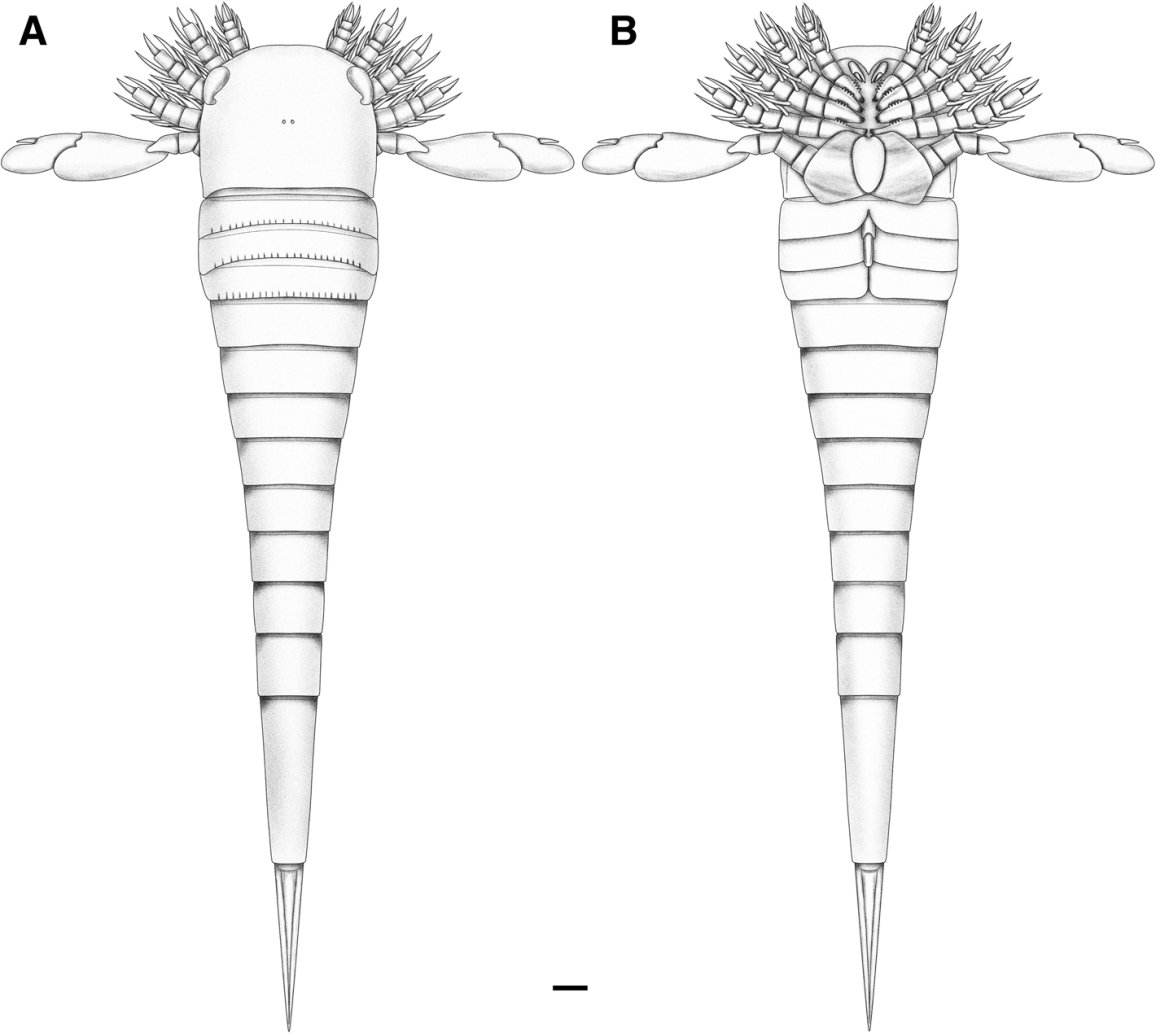
*Hoplitaspis hiawathai*, reconstruction. **a** Dorsal view. **b** Ventral view. The form of the first and second opercula are hypothetical and reconstructed based on comparison with *Octoberaspis*. Scale bar = 10 mm

*Hoplitaspis hiawathai* (Fig. 13) was an abundant, mid-sized predator. The robust prosomal appendages with their enlarged armature clearly indicate a predatory lifestyle, and closely resemble the appendage armature of the predatory eurypterid groups Carcinosomatoidea and Waeringopteroidea. The lateral eyes in *Hoplitaspis* are positioned similarly to those of predatory eurypterids, located marginally on the anterior of the prosomal shield. Although the lenses of *Hoplitaspis* are not preserved, studies on the visual systems of eurypterids have shown that taxa with marginally-positioned eyes have a higher visual acuity similar to that of modern predators [34, 35] while eurypterids with centrally-positioned eyes have a lower visual acuity akin to modern horseshoe crabs [36]. The lateral eyes of *Hoplitaspis* are curved around the prosomal shield margin (Fig. 5d), resulting in a broad field of view to the front of the animal that would have included a wide ventral arc. This indicates that *Hoplitaspis* may have preyed upon organisms on the sediment surface by dropping down upon them, a scenario supported by the enlarged swimming paddles of appendage VI.

The postabdominal articulations indicate that, as in eurypterids [37], *Hoplitaspis* was incapable of a great deal of lateral flexibility, although dorso-ventral flexing was possible (Figs. 4 and 6). This would have allowed the animal to rapidly change its orientation and position within the water column, but means that prey would have been subdued solely via the prosomal appendages, limiting the maximum prey size to have been smaller than that of *Hoplitaspis*. Despite the size of the appendage spines, the gnathobases of *Hoplitaspis* are relatively small and lack the enlarged coxal ‘teeth’ of eurypterids, indicating that prey items would not have had heavily sclerotized exoskeletons or calcium carbonate shells.

### Phylogenetic affinities

*Hoplitaspis hiawathai* most closely resembles the diploaspidid species *Dvulikiaspis menneri* [11], known from the Early Devonian of Siberia. The two species are similarly proportioned, with the postabdomen comprising the majority of the body length, being greater in length than the prosoma and buckler combined. The morphology of the buckler is also similar, being poorly differentiated dorsally from the postabdomen, with the second and third tergites having curved margins while the fourth tergite has a flattened posterior margin. *Hoplitaspis* and *Dvulikiaspis* also exhibit similarities in the paddle, which projects from underneath the prosomal shield close to the midsection.

A broader revision of the known chasmataspidid species is required before a full phylogenetic framework is developed; however, preliminary analysis of a few species as part of a broader phylogeny of chelicerates retrieved *Dvulikiaspis* as part of a polytomy with the Middle Devonian *Achanarraspis* and a clade of the remaining Devonian diploaspidids [1, 3]. *Loganamaraspis* resolves as the sister taxon to all diploaspidids, based predominantly on its supposed possession of a pediform appendage VI [7], although restudy of the only known specimen has been unable to confirm the morphology of the appendages (DJ Marshall pers. comm.; Lamsdell pers. obs.). The opisthosoma of *Loganamaraspis* closely resembles those of *Hoplitaspis* and *Dvulikiaspis*, with a short poorly-differentiated buckler and elongated postabdomen that comprises at least half the total body length. If the original description of the appendages of *Loganamaraspis* is accurate, *Hoplitaspis* would be phylogenetically closer to the Devonian diploaspidids due to the sixth appendage being expanded into a paddle, although this is highly conjectural given the uncertainty regarding the actual appendage structure of *Loganamaraspis*.

The exact phylogenetic position of *Loganamaraspis* aside, *Hoplitaspis* likely resolves as intermediate between *Chasmataspis* and the other diploaspidids. This inference is supported by the size of *Hoplitaspis*, which is similar in range to *Chasmataspis* [5, 6] and almost exponentially larger than all known Silurian and Devonian diploaspidids [1, 9–12, 18, 24, 38, 39], and the structure of the paddle (see ‘Implications for chasmataspidid evolution’).

### Elucidation of chasmataspidid morphology

*Hoplitaspis hiawathai* is the most complete chasmataspidid known, with portions of every aspect of its morphology demonstrated by multiple specimens. The new material affords a unique opportunity to study a chasmataspidid’s morphology in detail in part due to the large size of the species, resulting in individual aspects of morphology preserved in higher fidelity. This, combined with the variation in preservation among the specimens, allows for a number of long-standing questions about chasmataspidid morphology to be resolved. Critically, *Hoplitaspis* preserves complete prosomal appendages, evidence of the abdominal appendages, and details of the ventral buckler plate.

The exact number of podomeres in chasmataspidid prosomal appendages has been a matter of uncertainty. An isolated chelate appendage associated with *Chasmataspis* has eight podomeres [5], although it has been unclear which of the five locomotary appendage pairs it represents, with its possession of an exopod suggesting it represented appendage VI based on comparison with xiphosurans [6]. None of the previously described chasmataspidid species with paddles preserves a complete set of podomeres [11, 12, 18, 24], although one specimen of *Diploaspis casteri* preserves a partially disarticulated pediform appendage (likely appendage V based on its dimensions) consisting of eight podomeres [18, 24]. Understanding which limb pair these isolated, complete appendages belong to is complicated by the fact that many chelicerates exhibit a change in podomere count between the anterior and posterior appendages [40]. This is further complicated by comparisons with the closely related Eurypterida, which have nine podomeres in appendage V and VI [23, 40]. *Hoplitaspis* demonstrates that chasmataspidids also exhibit differential podomere counts between their appendages, with appendage II having seven podomeres and appendages III–VI having eight (Figs. 2, 9a, 8d and 9b). As such, the isolated *Chasmataspis* appendage may in fact belong to any of the third through sixth appendage pairs, although critically its possession of eight podomeres does not preclude it from being the sixth appendage, while the retention of the exopod (which is only found on appendage VI of modern xiphosurans [29] and may be homologous to the coxal ‘ear’ on appendage VI of Eurypterina [23]) strongly suggests that the *Chasmataspis* appendage may indeed be VI. Whether or not appendage VI of *Chasmataspis* was chelate, the eight-podomere paddle of diploaspidids cannot be considered homologous to the nine-podomere paddle of Eurypterina as evidenced by both structural differences and the occurrence of a plesiomorphically pediform appendage VI in Eurypterina [23, 31].

Chasmataspidid abdominal appendages have previously been described from the Devonian *Octoberaspis* [12] and Silurian *Loganamaraspis* [7]. Of these two taxa, *Octoberaspis* is the better preserved, with multiple specimens displaying the genital appendage and opercula while others preserve only the ventral buckler plate. Previously, researchers had considered chasmataspidids to lack abdominal appendages, with the ventral buckler plate representing a fused external surface [5, 24]. Tetlie and Braddy [7], when describing the ventral structures of *Loganamaraspis*, considered *Loganamaraspis* to have two opercula associated with the buckler, with the first operculum consisting of the fused appendages of the second and third segments and bearing a genital appendage. While the specimen clearly preserves a genital appendage consisting of two segments, only the third operculum is preserved and the anterior opercula are reconstructed based on comparison with eurypterids [7]. *Octoberaspis*, however, demonstrates that the three buckler opercula are unfused with the genital appendage inserting at the first operculum and extending to the posterior of the second [12]. *Hoplitaspis* preserves the occurrence of buckler opercula but not their configuration, and the genital appendage which again is short and comprises two segments. While *Hoplitaspis* does not preserve fine details of the abdominal appendages, their occurrence is important as it demonstrates that ventral buckler structures are present even though the majority of specimens only preserve the ventral buckler plate. It is possible that the high degree of sclerotization of the buckler plate in combination with the delicate structure of the abdominal appendages reduces the preservation potential of the opercula and genital appendages, and therefore the failure to preserve opercula and the genital appendage when a ventral plate is present should not necessarily be considered a true biological absence [41]. This has important implications for *Chasmataspis*, which only preserves a ventral plate [5] despite Cambrian trace fossils that appear to have been produced by *Chasmataspis*-like animals possessing three unfused buckler opercula and a genital appendage [6]. The buckler plate itself is considered likely to have formed the roof of a gill chamber in a manner similar to the fused sternites of the horseshoe crab thoracetron [26], with the opercula hanging from the ventral surface.

*Hoplitaspis* also preserves hitherto unknown details of the ventral buckler plate, particularly regarding its anterior margin. Despite being frequently observed, the ventral plate of *Diploaspis casteri* is not well preserved [18], while the ventral plates of *Chasmataspis* [5, 6] and *Octoberaspis* [12] do not clearly preserve the anterior of the buckler plate. *Hoplitaspis* demonstrates that the buckler plate has a bilobate anterior margin with a median notch (Figs. 8a, d and 9a), suggesting that the anteriorly-positioned, medially curving paired incisions described from the buckler plate of *Chasmataspis* may in fact represent the anterior of a partially displaced plate [5]. The ventral plate of *Hoplitaspis* bears a strong similarity to a structure in the Devonian chelicerate *Houia* that has been interpreted as an enlarged metastoma [3]. Originally considered a horseshoe crab [42], the discovery of a broad ventral plate with an anterior medial notch led to the interpretation of *Houia* as the sister taxon to a clade comprising chasmataspidids, eurypterids, and arachnids [3]. However, given the similarity between the plate in *Houia* and the ventral buckler plate of *Hoplitaspis* in combination with the fact that *Houia* has nine opisthosomal segments posterior to the plate, it is possible that *Houia* is a chasmataspidid.

### Implications for chasmataspidid evolution

Chasmataspidids have historically occupied a somewhat fluid position in chelicerate phylogeny, being considered either the sister group to Xiphosura [43–46], sister group to Eurypterida [46–48], in-group eurypterids [49], or sister group to a clade comprising Eurypterida and Arachnida [2–4, 50, 51]. While chasmataspidid monophyly has been generally accepted in recent years [2–4, 52], the group has also been considered to be paraphyletic to Eurypterida [7, 46] or polyphyletic [43–45]. A number of fundamental unresolved questions concerning chasmataspidid evolution have hampered attempts to resolve their phylogenetic affinities. Uncertainty over chasmataspidid monophyly has been driven by the distinctive, more xiphosurid-like morphology of *Chasmataspis* in comparison to the eurypterid-like morphology of diploaspidids [7, 43], concerns regarding the extreme size discrepancy between *Chasmataspis* and diploaspidids, and the suggestion that the expanded paddles of appendage VI in diploaspidids and Eurypterina may be homologous [47].

*Hoplitaspis* in many ways represents an intermediate form between *Chasmataspis* and diploaspidids, despite appearing in several ways unusual for a chasmataspidid, being a large active predator with a wide anterior field of view and enlarged raptorial appendages. This is most obvious in regard to the size of *Hoplitaspis*. All known Silurian and Devonian diploaspidids have total body lengths of < 30 mm, while the Ordovician *Chasmataspis* is substantially larger, with a body length of approximately 100 mm. *Hoplitaspis*, while possessing several diploaspidid characteristics, is also large (290 mm). This indicates that the origin of the diploaspidid morphology began during the Ordovician, and occurred prior to the decrease in size that otherwise characterizes the group.

The transitional nature of *Hoplitaspis* extends beyond body size. Silurian and Devonian diploaspidids possess a microtergite (the tergite of the first opisthosomal segment) that is strongly reduced, being 15–30% the length of the buckler tergites, and partially subsumed under the posterior of the prosomal shield [11]. In comparison, the microtergite of *Chasmataspis* is 50% the length of the subsequent buckler tergites and is not covered dorsally by the prosomal shield [5, 6]. *Hoplitaspis* possesses a larger microtergite than other diploaspidids, with a length 50–60% that of other buckler tergites, and is partially covered dorsally by the prosomal shield (Fig. 6), a morphology intermediate between that of *Chasmataspis* and other diploaspidids. *Hoplitaspis* also possesses antero-lateral extensions of the buckler, termed ‘shoulders’, that are present in diploaspidids [11] but absent from *Chasmataspis* [6]. However, the shoulders of *Hoplitaspis* lack the dorsal inflection evident in Silurian and Devonian diploaspidids [11], again marking the new species out as possessing an intermediate morphological condition.

**Fig. 14:**
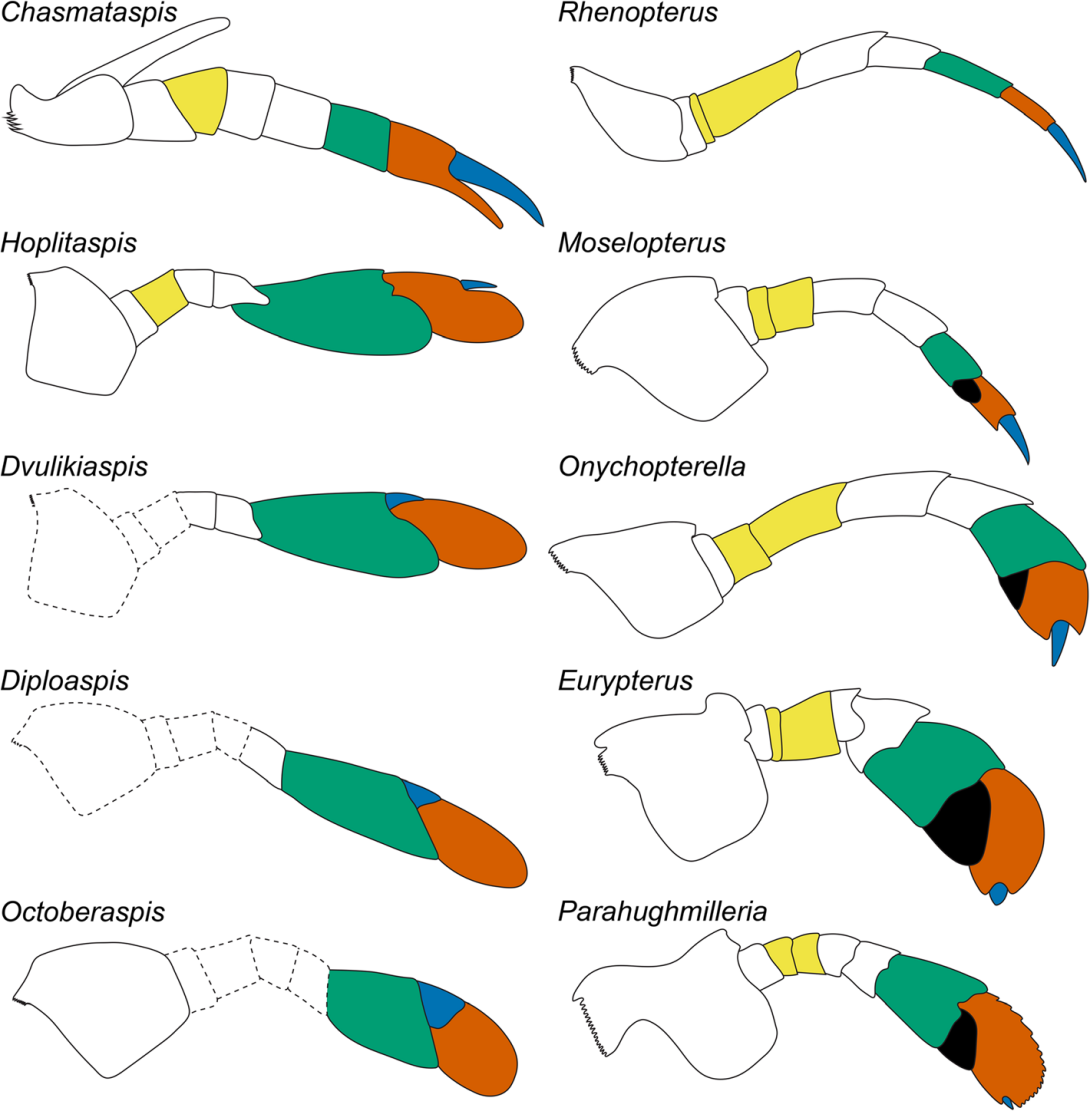
Chasmataspidid appendage VI podomere homology. Comparison of the morphology of appendage VI of the chasmataspidids *Chasmataspis*, *Hoplitaspis*, *Dvulikiaspis*, *Diploaspis*, and *Octoberaspis* and the eurypterids *Rhenopterus*, *Moselopterus*, *Onychopterella*, *Eurypterus* and *Parahughmilleria*. Proposed homology of the femur and distal podomeres are shown through color coding: yellow = femur (divided into the basifemur and telofemur in eurypterids), green = basitarsus, red = telotarsus, blue = apotele, black = ‘podomere’ 7a. Where the podomere morphology is unknown the reconstruction is shown by a dashed outline. The change in endopod insertion on the coxa in *Diploaspis* and *Octoberaspis* is inferred through the angle at which the paddle projects from underneath the prosomal shield and the in situ preservation of the coxa of *Octoberaspis*

The most instructive aspect of *Hoplitaspis*’ morphology is, however, the condition of appendage VI. *Chasmataspis* appears to have a distinct appendage structure as compared to diploaspidids, with a more xiphosuran-like chelate endopod [5, 6] rather than the eurypterid-like appendages of Diploaspididae [12, 18], with the likelihood that appendage VI was chelate in *Chasmataspis* conflicting with the expanded paddles in diploaspidids. While *Loganamaraspis* was originally interpreted as a diploaspidid with a pediform appendage VI [7], the evidence for this is not convincing and the details of the appendage morphology are at best unclear. *Hoplitaspis*, preserving a full complement of podomeres in appendage VI, permits an assessment of podomere homology between the chelate appendage of *Chasmataspis* and diploaspidid paddles for the first time (Fig. 14). Possessing eight podomeres in total, chasmataspidids are considered to possess an undivided femur in appendage VI based on phylogenetic bracketing with *Weinbergina* and Scorpiones, both of which exhibit the same undivided condition [40]. This has important ramifications for any potential comparison with eurypterids, as the division of the eurypterid appendage VI femur into the basifemur and telofemur means that the apoteles of chasmataspidids and eurypterids are still homologous, i.e. the eurypterid podomeres VI-5–VI-9 are equivalent to chasmataspidid podomeres VI-4–VI-8. *Hoplitaspis* indicates that the chasmataspidid paddle developed through the expansion of the seventh podomere, which comprised the fixed finger of the chelate appendage of *Chasmataspis*. This results in an elongate, relatively narrow paddle, a condition maintained in most diploaspidids [11, 18] with the exception of *Octoberaspis* [12], which develops a shorter, more robust paddle morphology. *Octoberaspis* also exhibits modifications to the anterior ‘podomere 7a’, which becomes thickened and quadrate. This segment is most likely misnamed; originally considered potentially homologous to the ‘podomere’ 7a of Eurypterina, which occurs on the ventral margin of the appendage distally from podomere 7 so as to overlap podomere 8, the structure in diploaspidids actually most likely originates at the distal margin of podomere 6. Crucially, any kind of podomere 7a is demonstrably absent from *Hoplitaspis*. However, podomere 8 is located midway along the dorsal margin of podomere 7, corresponding to the insertion point of the free finger of the chelate *Chasmataspis* appendage and rendering the paddle of *Hoplitaspis* technically (if not functionally) chelate. This suggests that the dorsal ‘podomere 7a’ of diploaspidids may actually be comprised of podomere 8, which could have migrated its insertion point proximally over the course of the evolution of the paddle (Fig. 14), while the eurypterid ‘podomere’ VI-7a has been hypothesized to be a modified ancillary spine [23]. With the exception of *Octoberaspis*, this structure tends to be narrow and triangular in diploaspidids [11, 18], a morphology fitting a spinous terminal podomere origin. While such an extreme dorsal migration of podomere 8 to the margin of podomere 6 may be unusual, it is not without precedent: in carcinosomatid eurypterids, podomere 7 articulates directly with podomere 5 along its dorsal margin [53, 54], while the fifth podomere apotele of the maxilliped forcipule of scolopendromorph and geophilomorph centipedes articulates directly with the second podomere through a dorsal pivot joint [55].

### Chasmataspidid biogeography

**Fig. 15:**
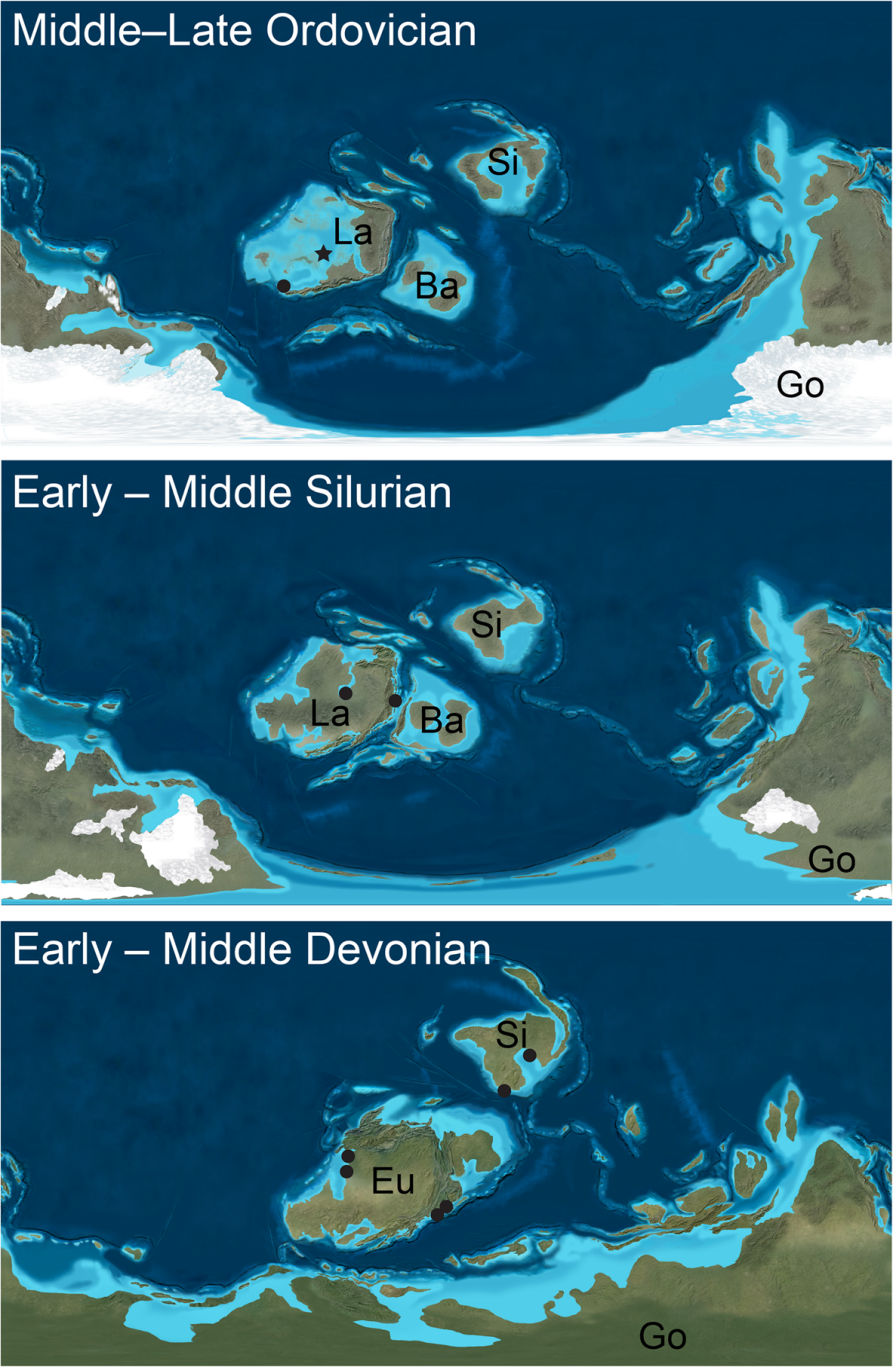
Geographic distribution of chasmataspidids. Occurrences are shown on paleogeographic reconstructions for the Middle–Late Ordovician, Early–Middle Silurian, and Early–Middle Devonian. *Hoplitaspis* is indicated by a star while other chasmataspidids are represented by circles. Ba = Baltica, Eu = Euramerica, Go = Gondwana, La = Laurentia, Si = Siberia. Paleogeographic reconstructions © Ron Blakey, Colorado Plateau Geosystems, used under license

The discovery of a second Ordovician chasmataspidid provides a test of the previously observed biogeographic pattern that, prior to the Devonian, chasmataspidids are known only from the paleocontinent of Laurentia [1]. *Hoplitaspis* fits with this proposed scenario, occurring in the shallow seas of western Laurentia (Fig. 15). It seems likely that chasmataspidids may have had their origins on the paleocontinent, with both known Ordovician species recorded from Laurentia, as well as the possible Cambrian chasmataspidid specimens [6]. Chasmataspidids do not appear to have been good dispersers, with the group only occurring in Baltica after its collision with Laurentia and spreading to Siberia in the Devonian after it comes into close contact with the combined continent of Euramerica (Fig. 15).

While the paucity of the chasmataspidid fossil record necessitates that these biogeographic hypotheses are tentative, it does seem likely that the majority of the group’s early evolution occurred on Laurentia, and that further discoveries in North America will aid our understanding of early chasmataspidid diversity and ecology. As it has been suggested that eurypterids may have originated in Gondwana and invaded Laurentia around the mid-Late Ordovician [56] it is interesting to note that chasmataspidid miniaturization appears to have occurred approximately contemporaneously with the radiation of eurypterids in Laurentia and Baltica. As *Hoplitaspis* demonstrates, chasmataspidids could fulfil the role of mid-sized predators that eurypterids grew to dominate in the Silurian and Devonian, and it is possible (although currently speculative) that competition between the two groups may have resulted in the extreme reduction in size within diploaspidids.

## Conclusions

The newly described chasmataspidid *Hoplitaspis hiawathai* from the Late Ordovician (Richmondian) Big Hill Lagerstätte of Michigan’s Upper Peninsula is the earliest reported diploaspidid and only the second chasmataspidid known from the Ordovician. *Hoplitaspis* reveals previously unknown aspects of chasmataspidid morphology, including a complete post-oral prosomal appendage podomere count, and provides further evidence of chasmataspidid abdominal appendage morphology. *Hoplitaspis* bridges the morphological differences between the Ordovician *Chasmataspis* and the Silurian-Devonian diploaspidids, possessing a prosoma and buckler morphology with strong affinities to that of the eurypterid-like diploaspidids while retaining some xiphosuran-like features prevalent in *Chasmataspis*. The structure of the paddle-shaped appendage VI in *Hoplitaspis* reveals that diploaspidid paddles are likely derived from a chelate morphology and are convergent with the paddles of Eurypterina, rather than being homologous. Critically, the anterior ‘podomere 7a’ of diploaspidids is most likely the spinous terminal eighth podomere that has migrated to the proximal margin of the expanded podomere 7.

*Hoplitaspis* was in many ways unusual for a chasmataspidid, being a large, active predator rather than the bottom-dwelling *Chasmataspis* or miniaturized diploaspidids. This unexpected diversity in ecological role suggests that the typical, small diploaspidid morphotype may not be representative of the group prior to the Silurian. The abundance of *Hoplitaspis* within the Big Hill fauna indicates it must have been an important component of the local Ordovician community, indicating chasmataspidids may have occupied a variety of roles within Laurentian ecosystems before the major radiation of eurypterids in the Late Ordovician. The discovery of *Hoplitaspis* supports previous observations of chasmataspidid biogeography, suggesting the group may have originated in Laurentia and radiated to other paleocontinents as they collided with Laurentia or came into close proximity. Therefore, further exploration of early Paleozoic Laurentian Lagerstätten may yet uncover new material with the potential to increase our knowledge of early chasmataspidid evolution.
