# Correction to: A common arthropod from the Late Ordovician Big Hill Lagerstätte (Michigan) reveals an unexpected ecological diversity within Chasmataspidida

**DOI:** 10.1186/s12862-021-01843-4

**Published:** 2021-06-07

**Authors:** James C. Lamsdell, Gerald O. Gunderson, Ronald C. Meyer

**Affiliations:** 1 Department of Geology and Geography, West Virginia University, 98 Beechurst Avenue, Brooks Hall, Morgantown, WV 26501 USA; 2 Middleton, USA; 3 Louiseville, USA

## Correction to: BMC Evol Biol (2019) 19:8 https://doi.org/10.1186/s12862-018-1329-4

Following the publication of the original article [1], it has been brought to the authors' attention that the rights to the minerals and fossils excavated at the site are federally owned by the US Forest Service and are not under the jurisdiction of the Board of Commissioners of Delta County, Michigan. Therefore, future research of the locality will require a federal permit.
